# Perfluorocarbon-polyepinephrine core-shell nanoparticles as a near-infrared light activatable theranostic platform for bimodal imaging-guided photothermal/chemodynamic synergistic cancer therapy

**DOI:** 10.7150/thno.102743

**Published:** 2025-01-01

**Authors:** Kyung Kwan Lee, Kyung-Woo Park, Sang Cheon Lee, Chang-Soo Lee

**Affiliations:** 1 Bionanotechnology Research Center, Korea Research Institute of Bioscience and Biotechnology (KRIBB), Daejeon 34141, Republic of Korea.; 2 Department of Maxillofacial Biomedical Engineering, College of Dentistry, Kyung Hee University, Seoul 02447, Republic of Korea.; 3 Department of Biotechnology, University of Science & Technology (UST), Daejeon 34113, Republic of Korea.

**Keywords:** theranostics, perfluorocarbon, polyepinephrine, Fenton reaction, bimodal imaging, cancer treatment

## Abstract

**Background:** Activatable multifunctional nanoparticles present considerable advantages in cancer treatment by integrating both diagnostic and therapeutic functionalities into a single platform. These nanoparticles can be precisely engineered to selectively target cancer cells, thereby reducing the risk of damage to healthy tissues. Once localized at the target site, they can be activated by external stimuli such as light, pH changes, or specific enzymes, enabling precise control over the release of therapeutic agents or the initiation of therapeutic effects. Furthermore, these nanoparticles can be designed to incorporate multiple therapeutic modalities, including chemotherapy, photothermal therapy (PTT), and chemodynamic therapy (CDT). This comprehensive approach facilitates real-time monitoring of treatment efficacy and allows for dynamic adjustments to therapy, resulting in more personalized and effective cancer treatments.

**Methods:** This study reports the synthesis of perfluorocarbon (PFC)-encapsulated fluorescent polyepinephrine (PEPP) nanoshells chelated with Fe^2+^ (PFC@PEPP-Fe) and explores their potential for bimodal imaging and synergistic combination therapy in cancer treatment. The cellular uptake, cytotoxicity, and *in vitro* therapeutic efficacy of PFC@PEPP-Fe were assessed using 4T1 breast cancer cells. *In vivo* bimodal imaging using fluorescence (FL) and ultrasound (US) was conducted after injection into 4T1 tumor-bearing balb/c nude mice. The synergistic anticancer effects of PFC@PEPP-Fe, combining CDT and PTT, were evaluated following 808 nm laser irradiation (1 W/cm²) for 5 min, with treatment outcomes monitored over a 14 days period.

**Results:** Both *in vitro* and *in vivo* studies demonstrated that PFC@PEPP-Fe enables effective bimodal imaging and exhibits substantial anticancer efficacy through the synergistic effects of PTT and CDT. Near-infrared (NIR) laser irradiation increased the temperature, enhancing the release of O_2_ and the production of H_2_O_2_, which in turn amplified the CDT effect. The combination of PFC@PEPP-Fe administration and NIR laser significantly reduced tumor volume, slowed tumor growth, and improved survival in 4T1 tumor-bearing mice, confirming the strong anticancer activity due to the PTT/CDT synergy.

**Conclusions:** As a multifunctional theranostic nanoparticle, PFC@PEPP-Fe not only enables cancer cell-specific US/FL bimodal imaging through the generation of microbubbles from its PFC core and fluorescent PEPP shells but also facilitates synergistic chemodynamic and photothermal therapeutic actions under NIR laser irradiation, which induces the self-supply of H_2_O_2_ and O_2_ within cancer cells.

## Introduction

Cancer, a significant global health challenge, has necessitated the development of several innovative and effective therapeutic strategies. Recently, numerous theranostic nanoparticle-based strategies, including chemodynamic therapy (CDT), photothermal therapy (PTT), photodynamic therapy, immunotherapy, and radiotherapy, have been developed for cancer treatment 1, 2. CDT, which is particularly promising, involves the conversion of converting hydrogen peroxide (H_2_O_2_) into toxic hydroxyl radicals (∙OH) via a Fenton/Fenton-like-reaction (Fe^2+^ + H_2_O_2_ → Fe^3+^ + ∙OH + OH^-^), which induces inducing tumor-cell death 3. PTT, with its high specificity and spatial-temporal selectivity, has also been proven to be effective for targeted cancer treatment by converting near-infrared (NIR) light into localized heat 4. Nevertheless, challenges such as insufficient ∙OH production in CDT and limited tissue penetration in PTT hinder their therapeutic application 5, 6.

Consequently, synergistic combination therapy, which integrates various therapeutic modalities such as CDT/PTT and photodynamic therapy/PTT, has gained prominence in cancer treatment due to its potential to overcome the limitations of individual treatment approaches 7, 8. Moreover, these theranostic nanoparticles enable diagnostic imaging using fluorescence (FL), magnetic resonance, and ultrasound (US) modalities, contributing to the development of effective cancer suppression strategies 9-11. Among these various methods, FL and US imaging exhibit particularly high clinical applicability owing to their noninvasiveness, real-time imaging ability, and cost effectiveness 12, 13. Phase-changeable perfluorocarbon (PFC) nanodroplets, which have recently gained prominence as US contrast agents, have been used to fabricate several effective multimodal imaging agents 14-16. PFC nanodroplets exhibit high colloidal stability, prolonged circulation, and efficient tumor vascular permeability 17.

In recent years, our research has focused on diverse theranostic systems for cancer imaging and therapy, particularly hybrid nanocarriers constructed from the complexation or mineralization of various inorganic species (Cu^+^, CaCO_3_, MnCO_3_, etc.) on diverse polymeric nanostructures 18-22. In this work, we aim to expand our design approaches to multifunctional theranostic nanoparticles applicable to activatable “OFF-ON” dual-modality imaging (US/ FL) and synergistic CDT/PTT treatment of cancers. From a diagnostic perspective, the combination of US and FL optical imaging offers the advantage of providing morphological information about the tumor through US, as well as enabling the detection and precise localization of tumors via FL. This dual-modality imaging approach not only enhances the accuracy of tumor detection but also enables real-time monitoring of therapeutic responses, thereby providing a comprehensive diagnostic framework. Therapeutically, the integration of CDT and PTT allows for achieving optimal therapeutic efficiency through endogenous redox reactions and external laser induction. Moreover, this combination displays the synergistic effects of CDT-induced oxidative stress and PTT-induced localized hyperthermia, effectively amplifying the therapeutic outcome while minimizing off-target effects.

This study describes the design and synthesis of Fe^2+^-chelated PFC-encapsulated polyepinephrine (PEPP) nanoshells (PFC@PEPP-Fe). Structurally, PFC@PEPP-Fe comprises a PFC core and a PEPP-Fe shell, where the FL of PEPP is effectively quenched by chelated Fe^2+^. PFC acts as a US contrast agent and O_2_ carrier, ensuring the self-supply of O_2_ and H_2_O_2_ for enhanced-CDT. Moreover, the PEPP-Fe shell facilitates FL imaging and PTT upon near-infrared (NIR) light absorption under endosomal pH conditions.

**Scheme 1:**
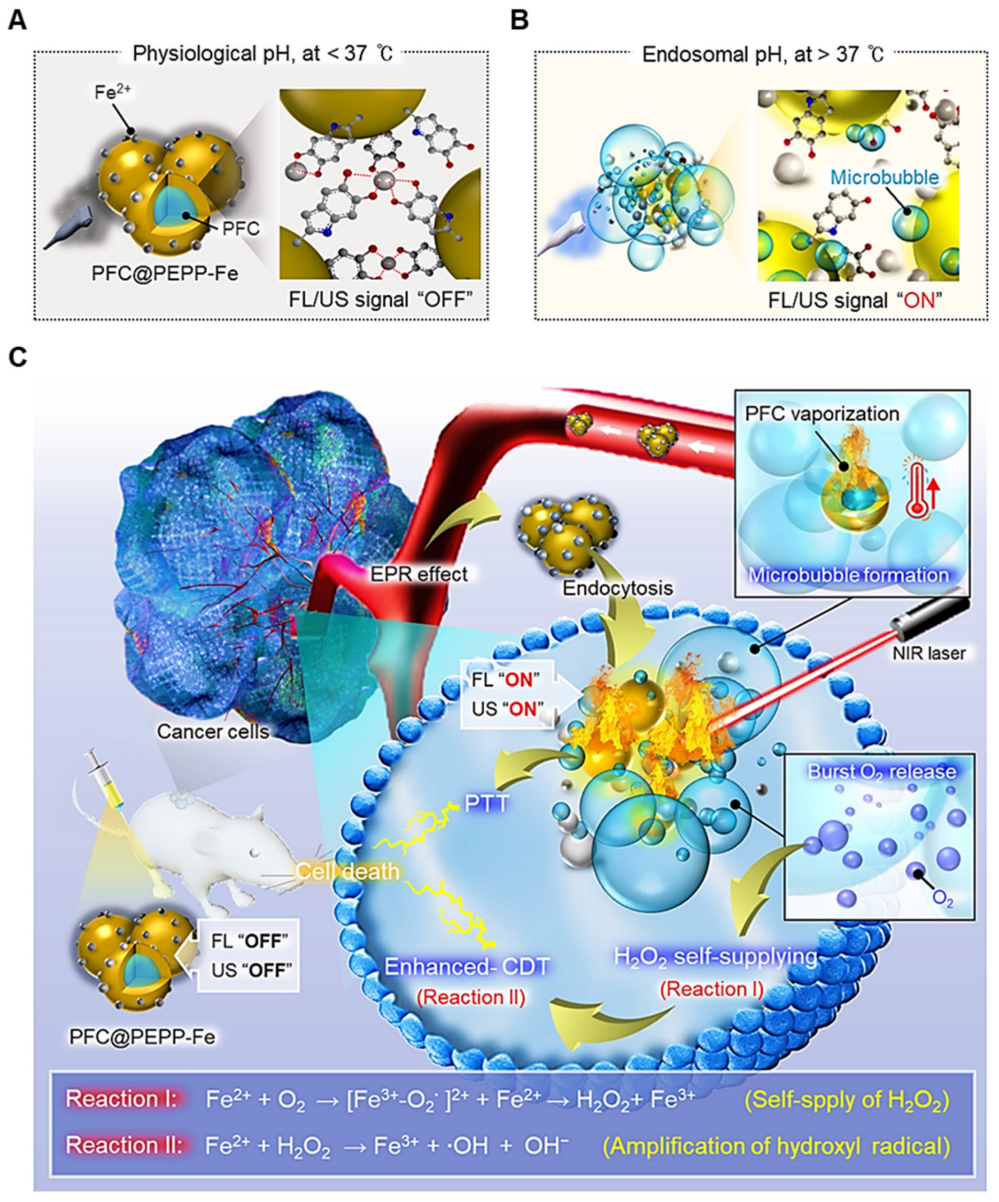
Scheme illustrating the working system of PFC@PEPP-Fe generating cancer-specific “OFF-ON” signals for activatable bimodal FL and US imaging at (A) physiological pH (below 37 ℃) and (B) endosomal pH (above 37 ℃). (C) Schematic illustration demonstrates the cancer treatment process of PFC@PEPP-Fe through combination of enhanced-CDT and PTT following cancer cell uptake facilitated by the EPR effect.

**Scheme 1a** and **Scheme 1b** show the working principle of PFC@PEPP-Fe, including the generation of activatable “OFF-ON” signals for US and FL and imaging. For US imaging, PFC@PEPP-Fe generated echogenic microbubbles via acoustic droplet vaporization of PFC at temperature above 37 ℃, generating cancer-cell-specific “OFF-ON” US signals. Upon internalization by cancer cells via the enhanced permeability and retention (EPR) effect, PFC@PEPP-Fe disassembled under acidic conditions in endosomes, resulting in the generation of cancer-specific FL signals by dissociation of Fe^2+^ from catechol-Fe complexes. Furthermore, NIR laser irradiation guided by cancer-specific dual-mode US/FL signals elevates temperature of cancer cells, results in generates of numerous gaseous microbubbles by the heat-induced phase transition of PFC, facilitating the release of O_2_. Subsequently, PFC@PEPP-Fe supplied H_2_O_2_ within cancer cells through the redox reactions of Fe^2+^ with O_2_, thereby representing an augmented combination of enhanced-CDT and PTT (**Scheme 1c**). The *in vitro* and *in vivo* proof-of-concept studies reported in this paper highlight the cancer-specific therapeutic potential of PFC@PEPP-Fe and confirm its high potential for dual-mode imaging and combination therapeutics.

## Results and Discussion

### Synthesis and structure characterization of PFC@PEPP-Fe

**Figure 1:**
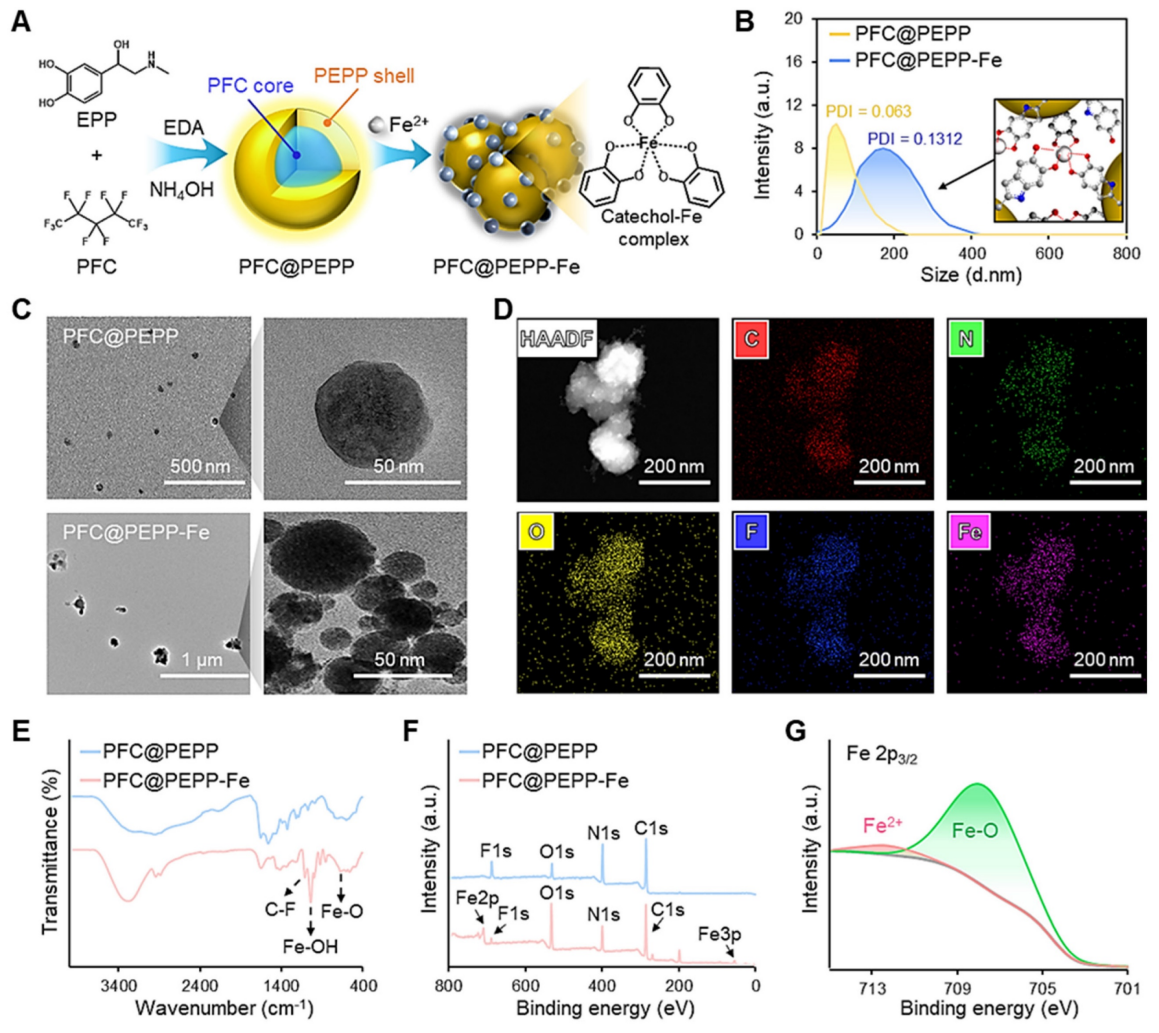
(A) Overall synthetic method of PFC@PEPP-Fe. Structure characterization of PFC@PEPP and PFC@PEPP-Fe using (B) DLS, (C) TEM, (D) TEM-associated EDS mapping, (E) FTIR, (F) XPS. (G) XPS narrow spectrum of PFC@PEPP-Fe includes specific peaks for Fe 2p.

The synthesis of PFC@PEPP-Fe involved the oxidation reaction of epinephrine (EPP), an analog of dopamine (DA), resulting in the polymerization of EPP into a polydopamine (PDA)-like structure (**Figure S1**) 23. **Figure 1A** depicts a simplified schematic illustration of the PFC@PEPP-Fe synthesis process. In this process, liquid PFC nanodroplets were encapsulated within PEPP nanoshells through the self-polymerization of EPP with ethylenediamine (EDA) in an alkaline solution. **Figure S2** shows the transmission electron microscopy (TEM) line scan image of PFC@PEPP, demonstrating its morphological structure, consisting of a PFC nanodroplet core and PEPP nanoshells. Subsequently, PFC@PEPP formed catechol-Fe²⁺ complexes on its surface through chelation, and we confirmed the loading of these Fe^2+^ ions through UV-Vis spectroscopy as well as a visible color change (**Figure S3** and **Figure S4**). As a result, PFC@PEPP-Fe has a larger average size (~180 nm) than does PFC@PEPP, along with a zeta-potential of 35.6 mV attributed to Fe^2+^ chelation (**Figure 1B** and **Figure S5**).

Morphologically, PFC@PEPP-Fe exhibited an agglomerated shape, while PFC@PEPP showed a narrow size distribution of spherical particles (**Figure 1C**), possibly due to the formation of catechol-Fe^2+^ complexes. The elemental composition of PFC@PEPP-Fe, including C, N, O, F, and Fe, was confirmed by mapping analyses using TEM-associated energy-dispersive X-ray spectroscopy (EDS) (**Figure 1D**). **Figure S6** illustrates the difference in the elemental composition between PFC@PEPP and PFC@PEPP-Fe by TEM.

Further investigation of the chemical bonding of PFC@PEPP-Fe was conducted using X-ray photoelectron spectroscopy (XPS) and Fourier-transform infrared (FT-IR) spectroscopy. The FT-IR spectra of both PFC@PEPP and PFC@PEPP-Fe showed peaks corresponding to the C-F bond (1062 cm^-1^) in PFC. In contrast, peaks corresponding to the Fe-OH (1033 cm^-1^) and Fe-O (611 cm^-1^) bonds in catechol-Fe were exhibited only by PFC@PEPP-Fe (**Figure 1E**) 24. **Figure S7** shows a reduction in the intensity of the peak corresponding to the C-OH bending (approximately 1350 cm^-1^) of catechol groups in PFC@PEPP upon the addition of Fe^2+^, providing further evidence that Fe^2+^ is chelated to the catechol group of PFC@PEPP 25. The XPS survey scan and elemental composition of PFC@PEPP and PFC@PEPP-Fe confirmed the presence of C, N, O, F, and Fe (**Figure 1F** and **Figure S8**). The Fe 2p spectrum exhibited peaks at 712.5 and 708.1 eV corresponding to Fe^2+^ and Fe-O, respectively, confirming the formation of catechol-Fe complexes in PFC@PEPP-Fe (**Figure 1G**) 26. A detailed XPS narrow-scan spectrum of PFC@PEPP-Fe, including the C1s, N1s, O1s, and F1s spectra, is shown in **Figure S9**; the spectrum contains distinct peaks at binding energies of 286.9, 285.2, 283.5, 399.7, 397.8, 531.5, 530.7, 529.7, 687.25, and 683.1 eV corresponding to C-F, C-O, C-C, NH_2_, pyrrolic N, Fe-OH, C-O, Fe-O, C-F_2_, and C-F_3_, respectively 27, 28.

### pH-Dependent FL changes and Fe^2+^ release of PFC@PEPP-Fe

**Figure 2:**
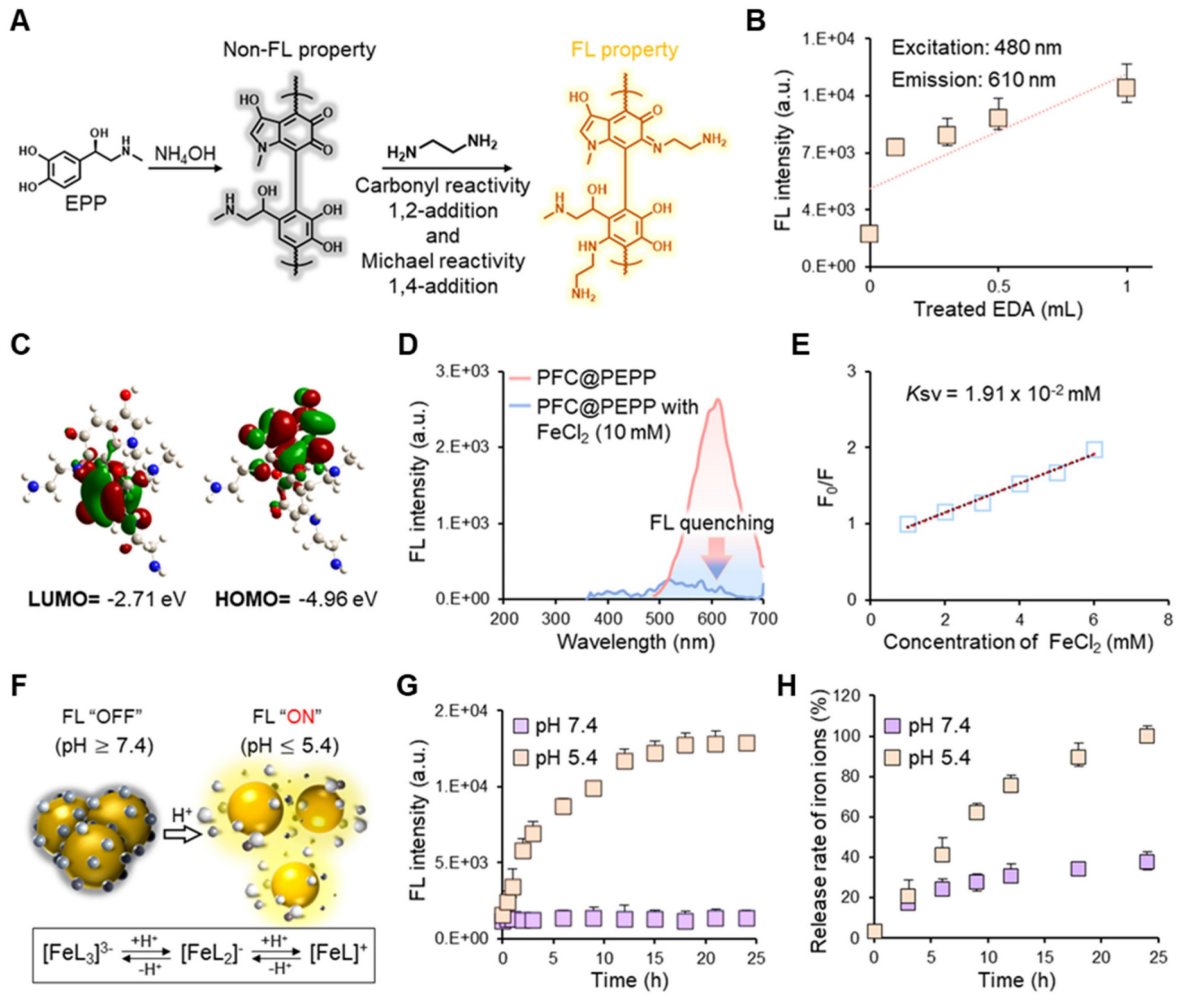
(A) Proposed synthetic mechanism and chemical structure of PFC@PEPP. (B) FL change of PFC@PEPP treated with different volumes of EDA. (C) Theoretically calculated molecular orbitals of PFC@PEPP using density functional theory calculations at the B3LYP/3-31G(d, p) level. (D) FL spectra of PFC@PEPP with/without Fe^2+^ ions. (E) Stern-Volmer plot of PFC@PEPP with different concentrations of FeCl_2_. (F) Schematic illustration of the FL “OFF-ON” working process of PFC@PEPP-Fe at different pH values. (G) FL change of PFC@PEPP-Fe in buffers with a pH of 7.4 and 5.4. (H) ICP-MS analysis of PFC@PEPP-Fe in pH 7.4 and pH 5.4 buffers at different time points.

To elucidate the optical properties of PFC@PEPP-Fe, we proposed the chemical structure of PFC@PEPP synthesized via the polymerization of EPP and EDA under mildly alkaline conditions through carbonyl 1,2-addition and Michael 1,4-addition reactions (**Figure 2A**) 29. Interestingly, we observed that EDA acts as an agent that imparts FL properties to PEPP by reducing the π-π stacking interactions within the PEPP structure (**Figure 2B** and **Figure S10**) 30. FT-IR and XPS analyses indicated an increase in peaks attributed to amine groups in PFC@PEPP with the addition of EDA, highlighting the significance of EDA incorporation in controlling the FL properties of PFC@PEPP (**Figure S11** and **Figure S12**) 31. Given by this, we calculated the energy levels of the molecular orbitals of PEPP (**Figure 2C**). As shown in **Figure S13**, fluorescent PEPP derived from EPP exhibited a narrower bandgap than that from fluorescent PDA synthesized from DA, which was previously studied by our group 32. The narrower band gap of PEPP results in excitation-dependent FL emission characteristics that are shifted closer to the NIR-I region (**Figure S14**). This finding supports our rationale that the use of PEPP as a PFC-decorating nanoshell is more suitable than PDA, for NIR light-responsive PTT applications.

Next, we examined the FL quenching properties of PFC@PEPP through Fe^2+^ chelation. Notably, PFC@PEPP demonstrated the ability to chelate various theranostic metal ions, such as Fe^2+^, Fe^3+^, Cu^2+^, and Pt^2+^, through catechol-metal coordination, leading to a significant decrease in FL (**Figure S15**). Nevertheless, we selected Fe^2+^ as the chelating ion for PFC@PEPP due to its efficient FL quenching effect and potential application as a Fenton-catalyst (**Figure 2D** and **Figure S16**). The FL quenching effect of Fe^2+^ on PFC@PEPP was analyzed using the Stern-Volmer equation, which revealed a decrease in FL reduction after FeCl_2_ (10 mM) addition (**Figure 2E** and **Figure S17**).

Subsequent investigations focused on the pH-dependent FL changes of PFC@PEPP-Fe to analyze controlled Fe^2+^ release under acidic conditions and verify the feasibility of the FL “OFF-ON” system. The proposed mechanism clarifies FL changes in response to alterations in the catechol-Fe complex structure and the Fe^2+^/Fe^3+^ concentrations across a broad pH range (**Figure 2F**) 33. Minimal changes in FL intensity were observed at physiological pH (pH 7.4), whereas significant FL recovery occurred within 3 h at pH 5.4, indicating disruption of the catechol-Fe complexes and Fe^2+^ release (**Figure 2G**). XPS analysis revealed changes in chemical bonding in PFC@PEPP-Fe under acidic conditions (pH 5.4), with increased Fe^2+^ intensity over time (**Figure S18**). Inductively coupled plasma mass spectrometry (ICP-MS) analysis confirmed that Fe^2+^ was released from PFC@PEPP-Fe under acidic conditions. As shown in **Figure 2H**, the PFC@PEPP-Fe exhibits a greater ability to release Fe^2+^ in a pH 5.4 buffer comprising sodium acetate (0.1 M) than in a pH 7.4 buffer comprising phosphate buffered saline (PBS). Consequently, after uptake by cells, PFC@PEPP-Fe is expected to generate a FL signal owing to the release of Fe^2+^ in the endosomal acidic conditions (**Figure S19**).

### *In vitro* FL and US imaging of PFC@PEPP-Fe

**Figure 3:**
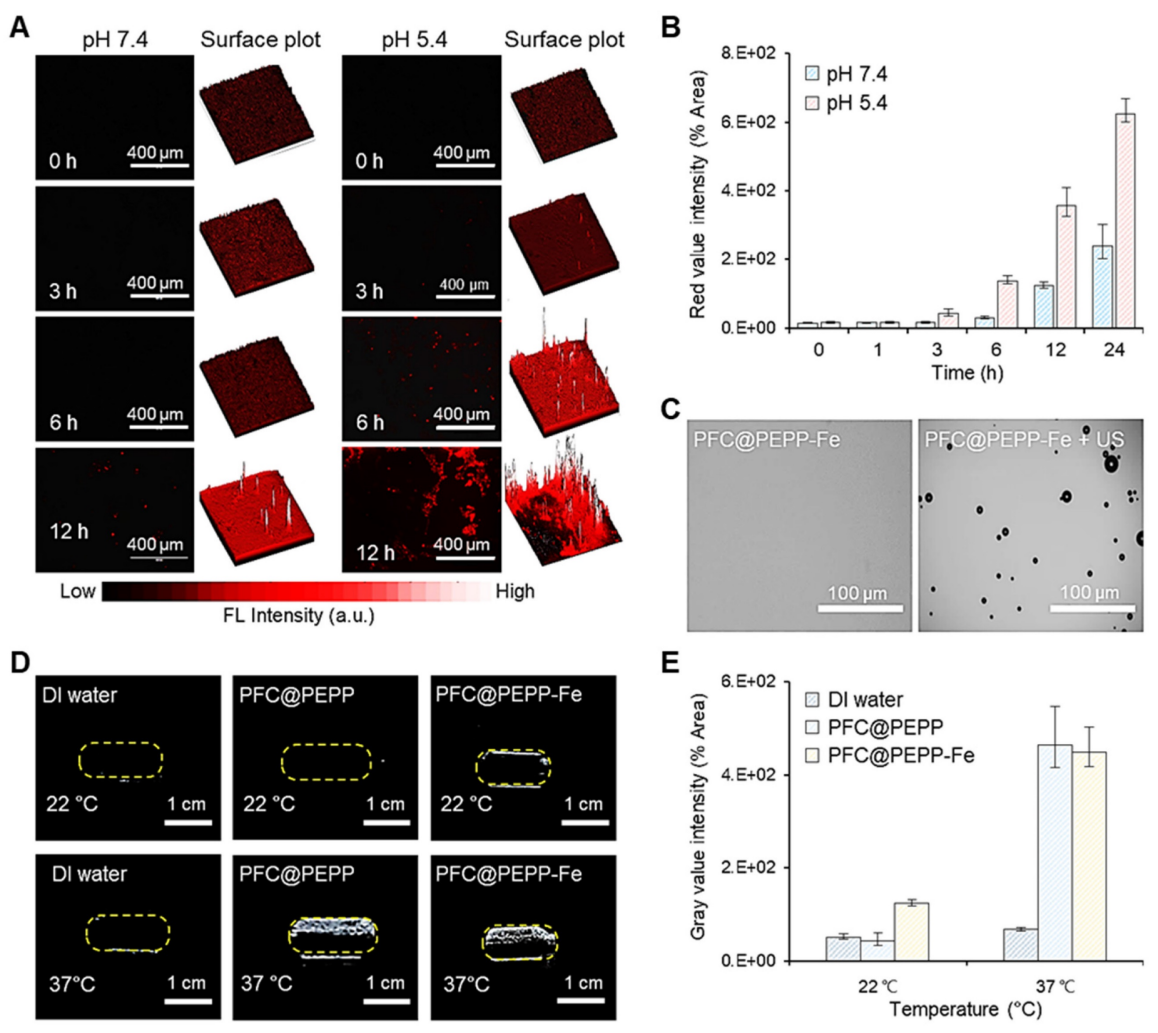
(A) Time-dependent FL microscopic images of 4T1 cells incubated with pre-dissolved PFC@PEPP-Fe solutions at pH 7.4 and 5.4, and the corresponding 3D surface-plot analysis of the FL signal. (B) Time-dependent quantitative FL intensity of 4T1 cells incubated with pre-dissolved PFC@PEPP-Fe solutions with pH = 7.4 and 5.4 (n = 3). (C) Optical microscopy images of PFC@PEPP-Fe with/without US exposure at 37 ℃ (US frequency: 10 MHz). (D) US images of PBS buffer and aqueous solutions of PFC@PEPP and PFC@PEPP-Fe at different temperatures and (E) their quantitative gray value intensities (n = 3).

Building upon these findings, we performed *in vitro* FL and US imaging of PFC@PEPP-Fe. The time-dependent FL images of 4T1 cells incubated with PFC@PEPP-Fe at different pH values (pH 7.4 and 5.4) for 12 h demonstrated rapid FL signal recovery at pH 5.4, indicating the disruption of catechol-Fe complexes and Fe^2+^ release (**Figure 3A** and **Figure 3B**). The US performance of PFC@PEPP-Fe was then investigated, demonstrating the phase-shift of PFC to gas under acoustic energy, leading to micron-scale bubble formation (**Figure 3C**). The frequency- and time-dependent US performances of PFC@PEPP and PFC@PEPP-Fe were explored (**Figure S20**, **Figure S21**, and **Figure S22**), revealing their suitability for US imaging at 37 ℃ but not at temperatures below 22 ℃, suggesting that acoustic energy-based US imaging can be performed at physiological temperature (**Figure 3D** and **Figure 3E**).

Expanding on these results, we now shift our focus to the heat-induced O_2_ release performance of PFC@PEPP-Fe. The high solubility of O_2_ in the PFC liquid phase enables both PFC@PEPP and PFC@PEPP-Fe, which contain a PFC core, to serve as reservoirs for O_2_ 34. Additionally, the low boiling point of PFC (29 ℃), found in both the PFC@PEPP and PFC@PEPP-Fe cores, facilitates O_2_ release upon heating, leading to microbubble formation through a liquid-gas phase transition of PFC 35, 36.

### NIR light-responsive temperature changes and O_2_ release from PFC@PEPP-Fe

**Figure 4:**
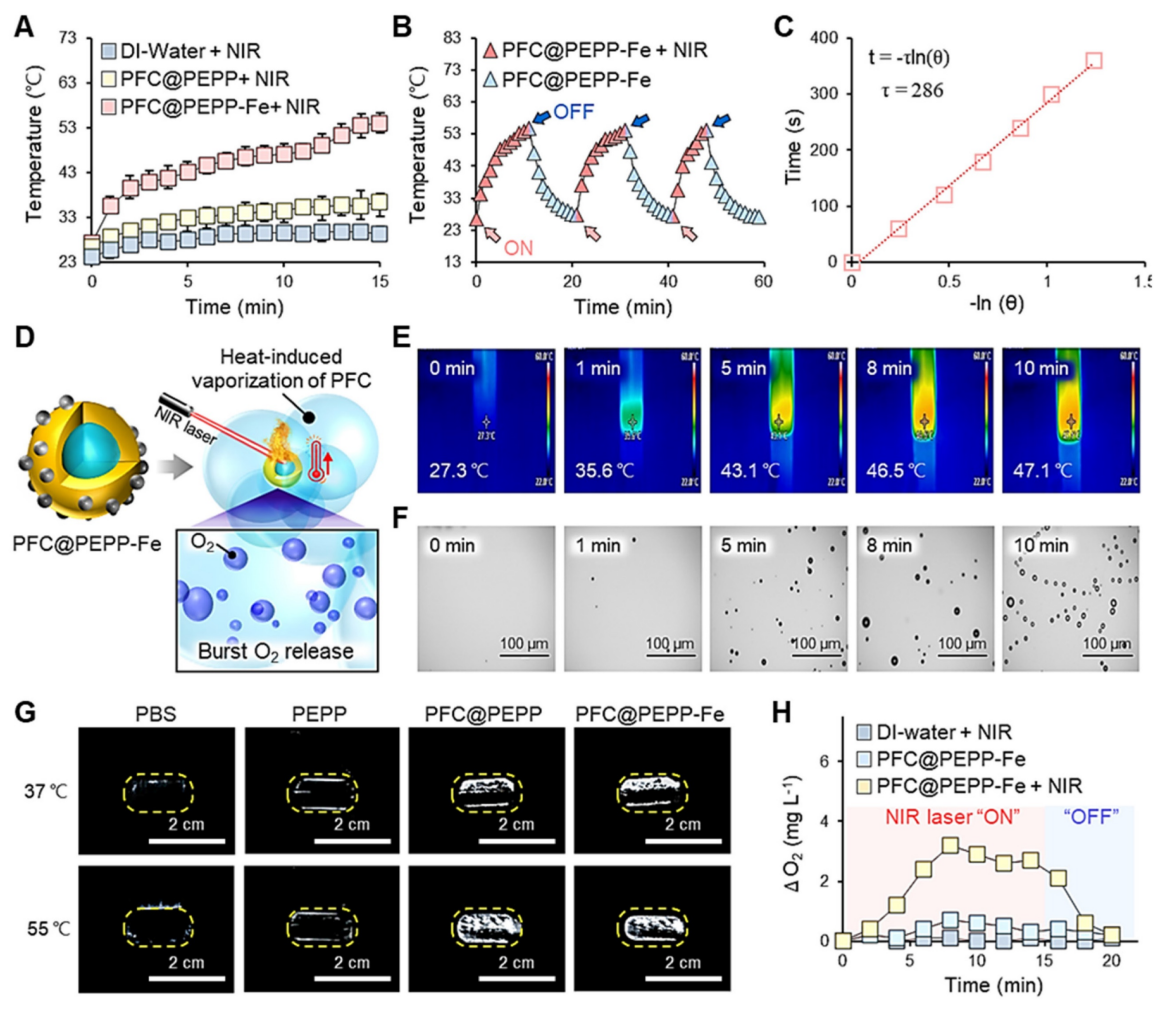
(A) Temperature variations of 1 mL of DI water and 1 mL of each aqueous solution of PFC@PEPP and PFC@PEPP-Fe under NIR laser irradiation (1 W cm^-2^). (B) Photothermal conversion stability of 1 mL aqueous solution of PFC@PEPP-Fe over three NIR laser irradiation (1 W cm^-2^) cycles. (C) Linear fit of the time vs. -ln(θ) plot of cooling-process data. (D) Schematics of the burst release of O_2_ from PFC@PEPP-Fe upon NIR laser irradiation. (E) IR thermal camera and (F) optical microscopy images of PFC@PEPP-Fe under continuous NIR laser irradiation for 10 min. (G) US imaging of PBS buffer and aqueous solutions of PEPP, PFC@PEPP, and PFC@PEPP-Fe at different temperatures. (H) Changes in the O_2_ concentration in aqueous solutions of PFC@PEPP and PFC@PEPP-Fe after NIR laser irradiation for 15 min.

**Figure 4A** displays the time-dependent temperature variations of PFC@PEPP (0.1 mg mL^-1^) and PFC@PEPP-Fe (0.1 mg mL^-1^), monitored separately using an infrared (IR) thermal imaging camera during continuous NIR laser irradiation (808 nm) for 15 min. Following 15 min of NIR irradiation exposure (1 W cm^-2^), the temperature of the aqueous solution of PFC@PEPP-Fe significantly increased from 27.3 to 55.2 ℃, while that of PFC@PEPP remained relatively unchanged, confirming the photothermal conversion effect under NIR laser irradiation in the former. This observation aligns with the UV-Vis absorption spectra of both sample groups; PFC@PEPP-Fe exhibits a broad absorption profile from the visible to the NIR region (300-800 nm), whereas PFC@PEPP shows negligible absorption in the NIR region (**Figure S23**). Subsequently, the temperature variations in aqueous solutions containing PFC@PEPP and PFC@PEPP-Fe upon exposure to different laser powers for 15 min were comprehensively assessed in real time (**Figure S24**). PFC@PEPP-Fe demonstrated excellent photostability, maintaining consistent performance over several NIR laser “ON-OFF” cycles (**Figure 4B**). Furthermore, the photothermal conversion efficiency (η) of PFC@PEPP-Fe was estimated to be ~19% (**Figure 4C**). Consequently, as depicted in **Figure 4D**, PFC@PEPP-Fe generates microbubbles when the temperature of the aqueous solution containing the sample increases upon exposure to NIR laser irradiation, potentially leading to the burst release of O_2_. **Figure 4E** and **Figure 4F** illustrate the temperature-dependent formation of microbubbles in the PFC@PEPP-Fe aqueous solution after 10 min of NIR laser irradiation. The laser-induced temperature increase in the PFC@PEPP-Fe solutions results in a gradual increase in microbubble formation, indicating a heat-induced phase transition within the PFC core of PFC@PEPP-Fe 37. **Figure S25** and **Figure S26** show temperature-dependent US images of PFC@PEPP and PFC@PEPP-Fe, respectively. As expected, both PFC@PEPP and PFC@PEPP-Fe generated significantly brighter US signals at 55 ℃ than at 37 ℃ due to the release of gaseous microbubbles with high impedance and compressibility by both species at 55 ℃ 38. In contrast, no US signal was observed for PEPP lacking a PFC core (**Figure 4G**). Thus, the PFC within the core of both PFC@PEPP and PFC@PEPP-Fe generates microbubbles via a heat-induced liquid-gas phase transition. To investigate the O_2_ release from the microbubbles generated by PFC@PEPP-Fe, the dissolved O_2_ concentration in aqueous solutions of PFC@PEPP-Fe was monitored in real time using a portable dissolved oxygen meter. As shown in **Figure 4H**, the dissolved O_2_ concentration in aqueous solutions of PFC@PEPP-Fe increased significantly upon exposure to NIR laser irradiation and subsequently decreased upon removal of the NIR laser irradiation source, confirming that the NIR laser-induced temperature increase in PFC@PEPP-Fe promoted microbubble formation, leading to the release of O_2_.

### Synergistic therapeutic efficacy of PFC@PEPP-Fe via CDT and PTT *in vitro*

To demonstrate the self-supplying H_2_O_2_ ability of PFC@PEPP-Fe and its amplification of ·OH, we assessed the radical generation capacity of PFC@PEPP-Fe *in vitro* using the TMB indicator. As shown in **Figure S27**, PFC@PEPP-Fe exhibited the highest radical generation capacity under acidic pH conditions with NIR laser irradiation, consistent with our hypothesis presented in **Scheme 1**.

**Figure 5:**
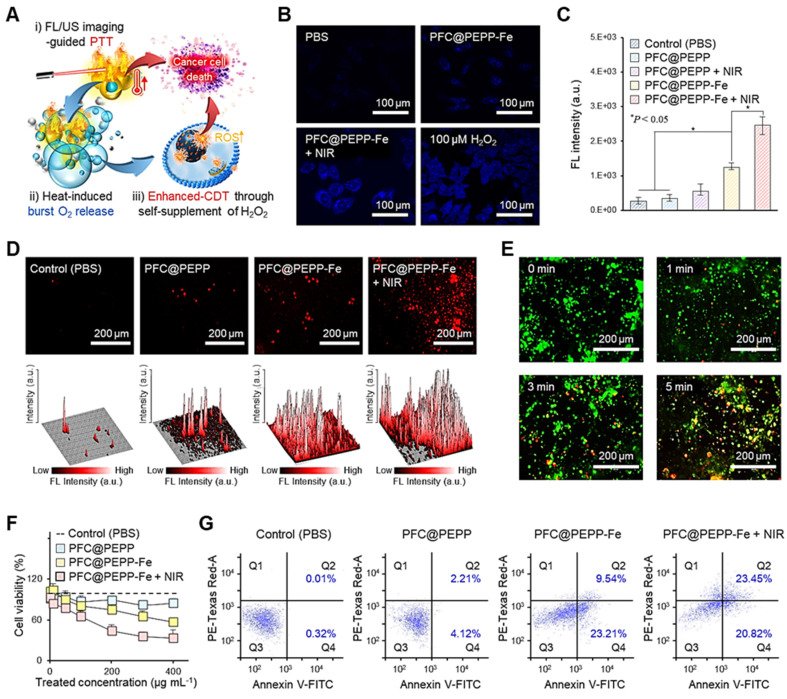
(A) Proposed therapeutic mechanism of PFC@PEPP-Fe for cancer treatment via dual-mode imaging-guided synergistic combination of PTT and enhanced-CDT. (B) FL images of 4T1 cells stained with H_2_O_2_ assay kit after treatment with various sample groups. (C) FL intensities of HTA in 4T1 cells treated with the various samples (n = 3). (D) Intracellular FL images of 4T1 cells treated with chemicals containing different concentrations of ROS (top) and the corresponding 3D surface-plot analyses of the FL signal (bottom). (E) FL images of 4T1 cells stained using a live/dead kit after treatment with PFC@PEPP + NIR. (F) Cell viability assessment of 4T1 cells treated with different concentrations of the various samples (n = 3). (G) Flow cytometry analysis of 4T1 cells treated with different sample groups.

Next, we examined the *in vitro* anticancer effect of PFC@PEPP-Fe. The proposed mechanisms, based on our hypothesis, and outlining the anticancer efficacy of PFC@PEPP-Fe via synergistic PTT and CDT, are shown in **Figure 5A**.

Upon internalization by 4T1 cancer cells, PFC@PEPP-Fe undergoes dechelation of catechol-Fe complexes in the acidic environment of endosomes, leading to the release of Fe^2+^ from PFC@PEPP in the system. Thus, this process produces tumor-specific FL signals. Additionally, NIR laser exposure for FL/US imaging triggered the burst release of O_2_ from PFC@PEPP-Fe within cancer cells, facilitating the self-supply of H_2_O_2_ through an O_2_ reduction mechanism, thereby producing extremely cytotoxic ·OH species through the Fenton-reaction (**Figure S28**) 39, 40.

To verify our hypothesis, we first confirmed the variations in H_2_O_2_ levels within cancer cells. As shown in **Figure 5B**, a high level of H_2_O_2_ was detected in 4T1 cancer cells treated with PFC@-PEPP-Fe + NIR group (comprising PFC@PEPP-Fe exposed to NIR laser irradiation), suggesting that PFC@PEPP-Fe + NIR can supply H_2_O_2_ within cancer cells. The fluorescent 2-hydroxyterephthalic acid (HTA) probe, formed by the reaction of terephthalic acid with ·OH, was employed for intracellular ·OH detection, with **Figure 5C** illustrating the detection of HTA fluorescence at excitation and emission wavelengths of 315 nm and 430 nm, respectively, in each experimental group. The PFC@PEPP-Fe + NIR group exhibiteed the highest FL intensity among all of the examined groups, confirming the intracellular amplification of ·OH through the self-supply of H_2_O_2_ under NIR laser irradiation. These results are consistent with the observed increase in the levels of reactive oxygen species (ROS), indicated by red FL, in 4T1 cells treated with PFC@PEPP-Fe + NIR (**Figure 5D**). Subsequently, the synergistic effect of PTT with PFC@PEPP-Fe was evaluated in 4T1 cells under NIR laser irradiation using live/dead cell staining and a cell counting kit-8 (CCK-8 assay). **Figure 5E** shows the live/dead evaluation results for 4T1 cells treated with PFC@PEPP-Fe and exposed to NIR laser irradiation for different durations. Significant red FL is observed in the 4T1 cells upon increasing the duration of NIR laser irradiation, indicating that photonic hyperthermia considerably improves the therapeutic effect of PFC@PEPP-Fe on tumor cells. Notably, 4T1 cells treated with the PFC@PEPP-Fe + NIR group (PTT and synergistic combination of CDT) exhibited greater reductions in cell viability than did those in the groups treated with only phosphate-buffered saline (PBS) (control), PFC@PEPP, or PFC@PEPP-Fe (CDT effect only) (**Figure 5F**). NIR laser-irradiated PFC@PEPP-Fe showed a pronounced anticancer effect on 4T1 cells characterized by notable morphological changes, including the loss of cell extensions, cellular rounding, and detachment (**Figure S29**). Furthermore, PFC@PEPP-Fe exhibits low cytotoxicity in normal cells while demonstrating significant anticancer effects in HeLa and MDA-MB-231 cells (**Figure S30**). Notably, PFC@PEPP-Fe demonstrates anticancer effects even without NIR laser irradiation, suggesting that this effect is attributed to ferroptosis induced by the release of iron ions (**Figure S31**).

Flow cytometry was used to investigate the anticancer efficacy of the synergistic combination of PTT and CDT. The flow cytometry results in **Figure 5G** indicate the effects of treating 4T1 cells with PBS (control), PFC@PEPP, PFC@PEPP-Fe (which exhibits the CDT effect only), or PFC@PEPP-Fe + NIR (which exhibits the synergistic combination of PTT and CDT). The percentage of apoptotic and necrotic cells was determined in each quadrant: Q1 (necrotic cells), Q2 (late apoptotic cells), Q3 (viable cells), and Q4 (early apoptotic cells). Treatment with PFC@PEPP-Fe and PFC@PEPP-Fe + NIR resulted in significant apoptosis in the Q2 and Q4 quadrants, indicating enhanced apoptotic cell death due to CDT (**Figure S32**). Notably, treatment with PFC@PEPP-Fe + NIR resulted in a greater percentage of necrotic cells with plasma-membrane rupture and eventual lysis in the Q1 phase than did treatment with PFC@PEPP-Fe, indicating that the high cancer-cell-killing efficacy of PFC@PEPP-Fe + NIR can be attributed to the synergistic effect of PTT and enhanced-CDT.

### *In vivo* FL and US imaging of PFC@PEPP-Fe

**Figure 6:**
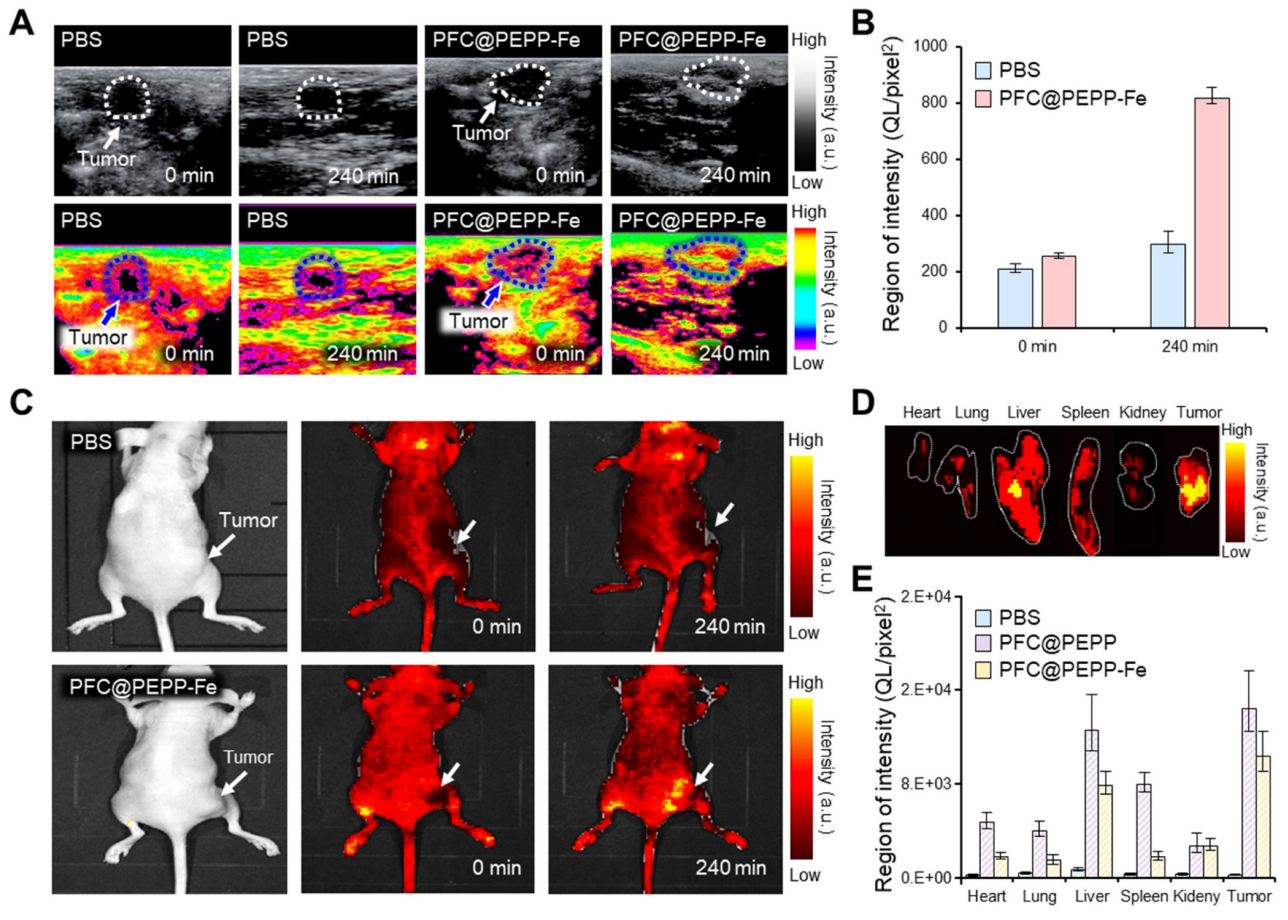
(A) US images of the tumor site (white and blue dotted line) in 4T1 tumor-bearing balb/c nude mice intravenously injected with PBS and PFC@PEPP-Fe, and (B) their quantitative intensities (n = 3). (C) FL images of 4T1 tumor-bearing balb/c nude mice intravenously injected with PBS and PFC@PEPP-Fe at 0 and 240 min. (D) FL image of the excised major organs and tumor of the mice after treatment with PFC@PEPP-Fe, and (E) their quantitative FL intensities (n = 3).

Considering the high biocompatibility and excellent *in vitro* FL/US imaging results, we investigated the *in vivo* dual-modality imaging and anticancer effects of PFC@PEPP-Fe. **Figure 6A** illustrates the US signal within tumor tissues of 4T1 tumor-bearing balb/c nude mice following intravenous administration of PBS and PFC@PEPP-Fe. In contrast to PBS, which does not cause detectable US contrast enhancement, the administration of PFC@PEPP-Fe results in a significant (over 3-fold) US-contrast enhancement within tumor tissues over time (**Figure 6B** and **Figure S33**).

Based on the *in vivo* US imaging results, the dual-modality capability of PFC@PEPP-Fe was verified using *in vivo* FL imaging. **Figure 6C** shows whole-body FL images of 4T1 tumor-bearing balb/c nude mice injected intravenously with PBS or PFC@PEPP-Fe. At the same time point as US imaging, an intense FL signal was observed in the tumor tissues of mice injected with PFC@PEPP-Fe compared with those of mice injected with PBS. The results obtained from the excision and examination of major organs and tumors demonstrated the selective targeting and efficient delivery of PFC@PEPP-Fe to tumor sites (**Figure 6D** and **Figure S34**). Notably, a biodistribution study after the intravenous administration of the samples confirmed that the FL intensity of PFC@PEPP in the liver and spleen was greater than that of PFC@PEPP-Fe, possibly because the size of PFC@PEPP-Fe (100-200 nm) enabled it to evade capture by Kupffer cells in the liver and splenic marginal zone macrophages in the spleen (**Figure 6E** and **Figure S35**) 41.

### Synergistic anti-cancer efficacy of the PFC@PEPP-Fe *in vivo*

**Figure 7:**
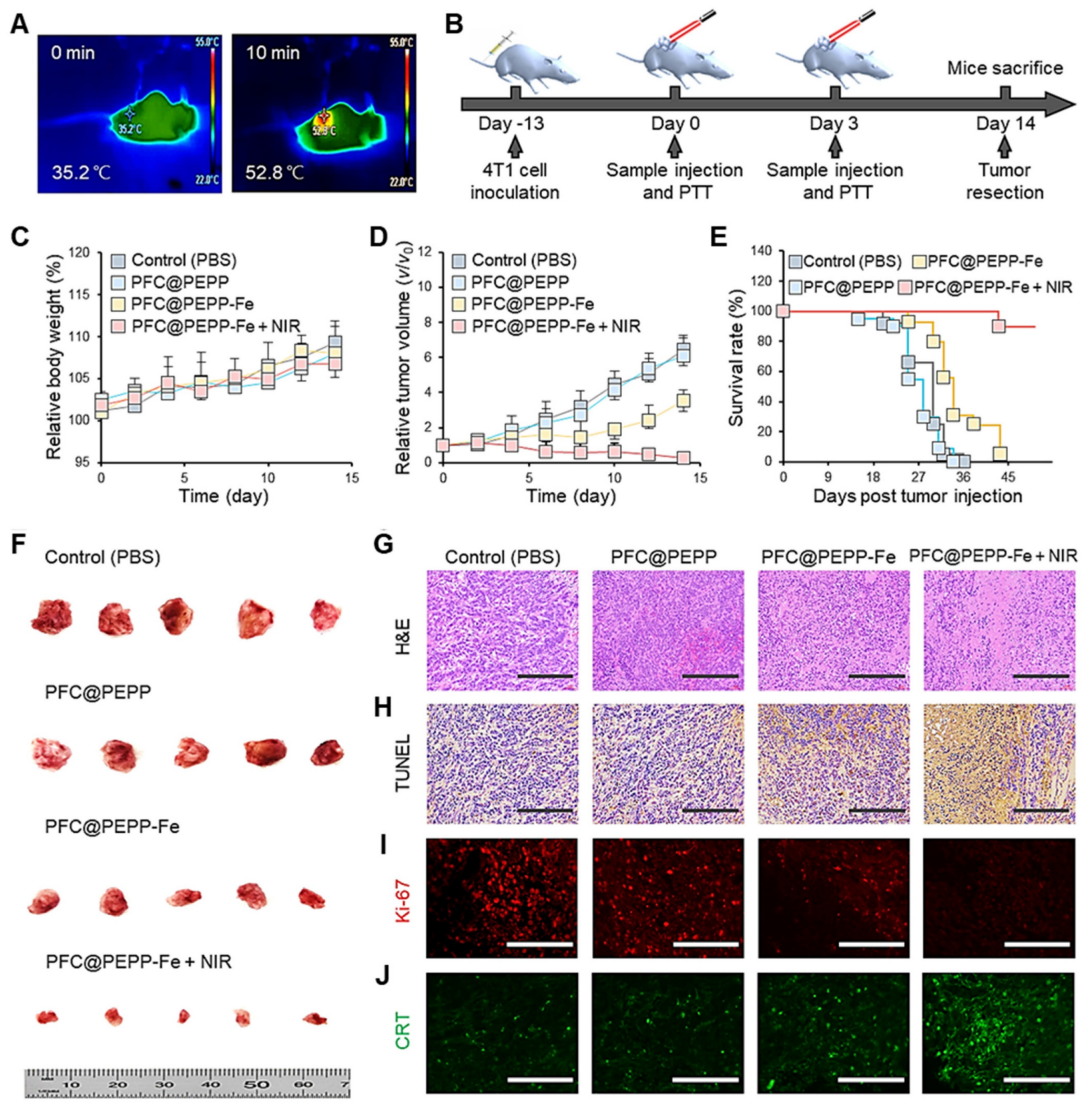
(A) IR thermal camera images of balb/c nude mice injected intravenously with PFC@PEPP-Fe under continuous NIR laser irradiation (1.0 W cm^-2^) for 10 min. (B) Schematics of the therapeutic schedule for 4T1 tumor-bearing balb/c nude mice. (C) Average body weight and (D) relative tumor volume of 4T1 tumor-bearing balb/c nude mice treated with only PBS, PFC@PEPP, PFC@PEPP-Fe, and PFC@PEPP + NIR laser irradiation. (E) Survival rate of the 4T1 tumor-bearing mice in the different treatment groups. (F) Camera images of excised tumors from the different groups after 14 days of treatment (n = 5). Histological evaluation of tumor tissues from the different treatment groups by (G) H&E and (H) TUNEL staining (scale bar = 200 μm). Histological evaluation of tumor tissues from the different treatment groups by (I) Ki-67 and (J) CRT immunohistochemical staining (scale bar = 100 μm).

Owing to the efficient delivery of PFC@PEPP-Fe to tumor tissues *in vivo*, the anticancer effect of PFC@PEPP-Fe was evaluated. **Figure 7A** shows the temperature changes in the tumor tissues of balb/c nude mice injected intravenously with PFC@PEPP-Fe recorded by an IR thermal camera after 10 min of NIR laser irradiation (1.0 W cm^-2^). NIR laser irradiation increased the temperature of the tumor tissue in mice to 52.8 ℃ within 10 min (**Figure S36**), which represents an effective hyperthermia temperature (>50 ℃) 42. Following an investigation of the biosafety of PFC@PEPP-Fe by monitoring changes in iron ion concentration in peripheral blood (**Figure S37**), a treatment schedule was designed comprising two intravenous injections of PFC@PEPP-Fe and two rounds of NIR laser irradiation over a 14 days period (**Figure 7B**). Throughout the experimental period, no significant changes in body weight were observed in any of the experimental groups (**Figure 7C**). However, the administration of PFC@PEPP-Fe and PFC@PEPP-Fe + NIR significantly reduced the tumor volumes (**Figure 7D**).

Notably, the PFC@PEPP-Fe + NIR group exhibited a significantly reduced tumor growth rate compared to that of the PFC@PEPP-Fe group (CDT effect only), highlighting the synergistic anticancer effect of PFC@PEPP-Fe + NIR attributed to PTT and CDT. Furthermore, the mice in the PFC@PEPP-Fe + NIR group exhibited a significantly longer survival period (>40 days) than did those in the other experimental groups (**Figure 7E**). Therefore, the *in vivo* anticancer efficacy of PFC@PEPP-Fe was enhanced by NIR laser irradiation owing to the synergistic effect of PTT and CDT. Images of the excised tumors corroborated the experimentally observed anticancer effects in the various groups (**Figure 7F**). Subsequently, owing to the effective anticancer efficacy of PFC@PEPP-Fe *in vivo*, hematoxylin and eosin (H&E) staining and a terminal deoxynucleotidyl transferase biotin-dUTP nick end labeling (TUNEL) assay were used to analyze the histopathological anticancer efficacy and biocompatibility of the PFC@PEPP-Fe. The group treated with PFC@PEPP-Fe + NIR, which involved synergistic PTT and CDT, exhibited greater tumor-tissue disruption, nuclear shrinkage, and necrosis than the other groups (**Figure 7G**). Microscopic examination after immunohistochemical staining indicated that PFC@PEPP + NIR group contained the highest number of TUNEL-positive cells exhibiting deoxyribonucleic acid damage among all of the experimental groups, confirming that it had the greatest degree of apoptotic activity (**Figure 7H**). Notably, as shown in **Figure 7I** and **Figure 7J**, the group treated with PFC@PEPP-Fe + NIR exhibited a significant reduction in the expression of the Ki-67 protein (red FL), a cancer marker, along with a substantial increase in the expression of calreticulin (CRT) protein (green FL), a marker of immunogenic cell death. These results indicate that PFC@PEPP demonstrated strong anticancer efficacy through enhanced-CDT. To assess the potential *in vivo* toxicity of PFC@PEPP-Fe, the major organs (heart, liver, spleen, lungs, and kidneys) of balb/c nude mice subjected to PFC@PEPP and PFC@PEPP-Fe treatment for 14 days were analyzed by H&E staining. The negligible major-organ damage and adverse effects in both the PFC@PEPP and PFC@PEPP-Fe + NIR groups confirmed the low toxicity of both samples (**Figure S38**).

## Conclusions

In summary, this study reports PFC@PEPP-Fe, a theranostic nanoparticle with promising applications for dual-mode imaging and synergistic combination therapy of cancers. The as-synthesized theranostic nanoparticles showed tumor-specific US imaging capabilities owing to microbubble generation via the acoustic droplet vaporization of PFC at physiological temperature, and cancer cell specific “OFF-ON” FL signals owing to the chelation of Fe^2+^ with the catechol groups in PFC@PEPP.

*In vitro* and *in vivo* studies confirmed the dual-mode (FL/US) imaging capability of PFC@PEPP-Fe, which exhibited excellent anticancer efficacy owing to the synergistic effect of PTT and CDT. NIR laser irradiation of PFC@PEPP-Fe led to an increase in temperature, which enhanced the release of O_2_ and the production of self-supplied H_2_O_2_, thereby enhancing the CDT effect in the system. The combination of PFC@PEPP-Fe administration and NIR laser irradiation significantly suppressed tumor volume and the tumor growth rate in 4T1 tumor-bearing balb/c nude mice and improved overall survival, confirming the high anticancer activity of PFC@PEPP-Fe owing to PTT/CDT synergism.

As a theranostic platform, the general applicability of our system can be expanded by the diverse choice of anchored Fenton or Fenton-like catalysts, including Fe^2+^, Cu^+^, Mn^2+^, Co^2+^, Ce^3+^, Cr^3+^, and Ru^3+^. Each metal ion may play a pivotal role by enabling PFC@PEPP to modulate therapeutic activity as well as activatable imaging performance.

Overall, this study confirms the high potential of PFC@PEPP-Fe as a theranostic platform for dual-mode imaging and combination therapy. With its unique applicability for both imaging and targeted therapy, PFC@PEPP-Fe holds promise for revolutionizing cancer treatment and significantly improving patient outcomes; however, further clinical evaluations across various cancer types are warranted. Nevertheless, the results of this study could guide the development of innovative nanocarriers and provide valuable insights into the design of high-performance theranostic systems in the future.

## Materials and Methods

### Materials

Ammonium hydroxide solution (28%), (±)-EPP hydrochloride, EDA (99.5%), terephthalic acid (TA) (97%), ethyl alcohol (99.5%), iron(II) chloride, and 3,3′,5,5′-tetramethylbenzidine (TMB) were purchased from Sigma-Aldrich (Milwaukee, WI, USA) and used without further purification. Perfluoro-n-pentane (C_5_F_12_) was purchased from Stem Chemicals (Newbury, USA). Dialysis-membrane bags (molecular weight cutoff (MWCO): 10 kDa), Roswell Park Memorial Institute (RPMI) 1640 medium, fetal bovine serum (FBS), trypsin-EDTA solution, PBS, CellROX Deep Red live/dead cell staining kits, Annexin V-fluorescein isothiocyanate (FITC), PE-Texas Red-A (PE), and penicillin/streptomycin (P/S) were purchased from Thermo Fisher Scientific Inc. (Waltham, MA, USA). A hydrogen peroxide assay kit (Cell Meter^TM^) was purchased from ATT Bioquest (California, USA). The 4T1 cells used for experimentation were obtained from the American Type Culture Collection (ATCC) (USA). CCK-8 and Lipid Peroxidation Assay Kit (BDP 581/591 C11) were purchased from Dojindo Co. Ltd. (Tokyo, Japan).

### Synthesis of PFC@PEPP-Fe

PFC@PEPP-Fe nanocarriers were synthesized using a facile self-polymerization method. Initially, a mixture of C_5_F_12_ (120 µL) and DI water (2.7 mL) was prepared in a 5-mL amber glass vial by ultrasonication (Young Jin Corporation, Daejeon, South Korea, 130 W) at an amplitude of 10% for 2 min. Subsequently, a mixture comprising ethanol (1.2 mL) and an ammonium hydroxide solution (120 µL) was added to the PFC solution. After stirring this solution for 30 min, an aqueous solution comprising EPP hydrochloride (15 mg) and EDA (1 mL) was added to initiate polymerization. Subsequently, the solution was stirred for 24 h and dialyzed in DI water for 3 days to remove impurities. The resulting solution was centrifuged at 12000 rpm for 20 min. Prior to surface modification, freshly prepared PFC@PEPP was suspended in DI water (1 mg mL^-1^) followed by the addition of FeCl_2_ (10 mM), and it was stirred for 24 h. The as-synthesized PFC@PEPP-Fe was washed thrice by centrifugation, and the supernatant was replaced with DI water for further analysis.

### Structure characterization

PFC@PEPP and PFC@PEPP-Fe were structurally characterized using FT-IR (Bruker Corp., Billerica, MA, USA) and XPS (Thermo-Fisher Waltham, MA, USA). The morphological shapes and atomic compositions of PFC@PEPP and PFC@PEPP-Fe were analyzed using TEM (G2 F30X-TWIN, 300 kV, USA). The hydrodynamic diameter and zeta potential of PFC@PEPP and PFC@PEPP-Fe were determined using a particle size analyzer (Malvern Zetasizer Nano ZS, UK).

### Theoretical calculations

The energy levels of the molecular orbitals of PEPP and PFC@PEPP were calculated using DFT with Gaussian 16. The molecular orbitals of the possible moieties were imported at the B3LYP/6-31G(d, p) level of theory.

### Optical properties

The FL spectra of PFC@PEPP and PFC@PEPP-Fe were recorded by an FL spectrometer (FluoroMate FS-2, Scinco, South Korea). The FL quenching of PFC@PEPP by Fe^2+^ was estimated using the Stern-Volmer equation.

*F*_0_/*F* = 1 + *K*_SV_ [Q]

In this equation, *F*_0_ and *F* indicate the FL intensities of PFC@PEPP at 610 nm in the absence and presence of Fe^2+^, respectively. *K*_SV_ is the Stern-Volmer quenching constant and [Q] indicates the concentration of the quencher (Fe^2+^).

### pH-Dependent Fe^2+^ release

ICP-MS (OPTIMA 7300 DV, Perkin-Elmer, USA) was used to quantify the release of Fe^2+^ from PFC@PEPP-Fe. During experimentation, PFC@PEPP-Fe (1 mL, 1 mg mL^-1^) was immersed in acetate buffer (10 mL, 0.1 M, pH 5.4) and PBS (0.1x, pH 7.4) in a dialysis-membrane bag (MWCO: 10 k). Subsequently, the original buffers were replaced with fresh PBS and acetate buffer at predetermined time intervals, and the replaced buffer samples were analyzed by ICP-MS.

### Visualization of the cellular uptake

In this study, 4T1 cells were cultured in 100-mm dishes with RPMI-1640 cell culture medium supplemented with FBS (10%) and P/S (1%). The cells were incubated in a humidified incubator at 37 ℃ with CO_2_ (5%) and subcultured using a trypsin-EDTA solution when they reach a confluency of ~80%. To visualize the pH-dependence of the intracellular FL signals, PFC@PEPP-Fe (0.1 mg mL^-1^) was dissolved in PBS (0.1x, pH 7.4) and acetate buffer (0.1 M, pH 5.4) for 12 h, and 10 μL of each solution was added to 4T1 cells cultured in a 96-well plate at a concentration of 5.0 × 10^3^ cells per well. After incubation for 12 h, the cells were washed twice with PBS, and intracellular FL images were recorded using an EVOS FL cell imaging system (Thermo Fisher, Waltham, MA, USA).

### *In vitro* US imaging

A color Doppler ultrasonic diagnostic apparatus (Canon Medical Systems, USA) was used for *in vitro* US imaging. Prior to imaging, a PFC@PEPP-Fe solution was loaded into a plastic dropper and subjected to US-probe detection at different temperatures, time intervals, and frequencies. The acquired *in vitro* US images were quantified using the Multi-Gauge Software (Fuji Photo Film Co. Ltd. Japan).

### *In vitro* photothermal conversion efficiency

The absorbances of PFC@PEPP and PFC@PEPP-Fe were measured using a UV-Vis spectrophotometer (Beckman Coulter, DU 800). The real-time temperature changes in PFC@PEPP were investigated by subjecting aqueous solutions containing the specimen to NIR light irradiation for 15 min. The photothermal stability of PFC@PEPP-Fe was evaluated through three cycles of “ON-OFF” control, each comprising 15 min of irradiation by an 808 nm laser. An IR thermal camera (GTC 400, BOSCH, Germany) was used to determine the η of the as-synthesized samples.

The η value of PFC@PEPP-Fe was calculated using the following equation from the literature:



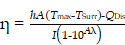



Here, *h*, *A*, *T*_max_, *T*_Surr_, *Q*_Dis_, *I*, and *A*λ represent the heat-transfer coefficient, surface area of the container, equilibrium temperature, ambient temperature, heat dissipation from absorbed light, incident laser power, and absorbance of PFC@PEPP-Fe at 808 nm, respectively. The *hA* value was calculated using the following equation:


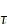
_s_ = 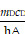


Here, τ_s_ indicates the sample-system time constant, while *m*_D_ and *c*_D_ indicate the mass and heat capacity of the DI water used as the solvent, respectively.

### Measurement of O_2_ release

The O_2_ concentration (mg L^-1^) in DI water was measured using a dissolved oxygen meter (VSTAR30, Thermo Fisher Scientific, USA) before and after exposure to (I) NIR laser irradiation (808 nm) and before and after the addition of (II) PFC@PEPP-Fe and (III) PFC@PEPP-Fe + NIR laser (808 nm). An NIR laser with a power of 1 W cm^-^² was used for irradiation.

### TMB analysis

To assess the radical amplification performance of the PFC@PEPP-Fe-mediated TMB colorimetric reaction, various groups of PFC@PEPP-Fe were dispersed in PBS buffer (0.1x, pH 7.4) and acetate buffer (0.1 M, pH 5.4) containing TMB (0.5 mM) and/or H_2_O_2_ (1.0 M) in test tubes, with or without 1 min NIR laser irradiation (1.0 W cm⁻²). Following a 30 min incubation, each test group was transferred into a 96-well plate, and absorbance was measured at 650 nm using a microplate reader.

### Evaluation of intracellular H_2_O_2_, ∙OH, and ROS levels

A Cell Meter™ was used to quantify the H_2_O_2_ levels in live 4T1 cells. The 4T1 cells were seeded at a density of 1.0 × 10^4^ cells per well in 96-well plates and divided into 4 groups. The cells were then stained with the working reagent for 30 min. After staining, each group was washed twice with PBS and subsequently incubated with PBS (control), PFC@PEPP-Fe, PFC@PEPP-Fe + NIR laser, or H_2_O_2_ (100 μM) for 6 h. Following incubation, the FL images of each group were measured using EVOS.

Lipid peroxidation probe was used for detecting lipid peroxidation in 4T1 cells. The 4T1 cells were seeded at a density of 2.0 × 10^4^ cells per well in 8-well plates, and PFC@PEPP-Fe (0.1 mg mL^-1^) were treated and cultured for 12 h, then the BDP 581/591 C11 working solution was added to each well according to the manufacturer's protocol. The FL images of each group were measured using EVOS.

To quantify ∙OH and visualize ROS levels in live 4T1 cells, HTA and CellROX Deep Red (excitation wavelength: 644 nm, emission wavelength: 665 nm) were utilized. For ∙OH quantification, 4T1 cells were seeded at a density of 1.0 × 10^4^ cells per well in 96-well plates and incubated with PBS (control), PFC@PEPP, or PFC@PEPP-Fe for 6 h. After incubation, the cells were washed twice with PBS, and their FL intensities were recorded using a microplate reader (SpectraMax M2, Molecular Devices). For ROS visualization, 4T1 cells were seeded at a density of 3.0 × 10^4^ cells per well on 8-chamber culture slides and treated with PBS (control), PFC@PEPP, or PFC@PEPP-Fe for 6 h. After incubation, the cells were washed three times with PBS and fixed with 4% paraformaldehyde for 15 min. The fixed cells were then analyzed using an EVOS FL cell imaging system. To quantify and visualize the levels of H_2_O_2_, ∙OH, and ROS in 4T1 cells following NIR laser irradiation, the cells were irradiated with a laser power of 1 W cm^-2^ for 5 min.

### *In vitro* therapeutic effect

During experimentation, 4T1 cells (5.0 × 10^3^ cells well^-1^) were seeded into 96-well culture plates and incubated at 37 ℃ in a humidified incubator under CO_2_ (5%) for 24 h. After incubation, a solution of PFC@PEPP-Fe in RPMI-1640 medium (100 μg mL^-1^) was added to the wells for 6 h. Subsequently, the cells were exposed to an NIR laser (808 nm) with a laser power of 1 W cm^-^² for 5 min, and the cell viability was assessed using a live/dead cell staining kit according to the manufacturer's instructions; 4T1 cells (5.0 × 10^3^ cells per well) in a 96-well plate were used for the cell viability assay. The 4T1 cells were treated with different concentrations of PFC@PEPP (10, 50, 100, 200, and 300 μg mL^-1^) and incubated at 37 ℃ under CO_2_ (5%). After 24 h of incubation, the CCK-8 reagent was added to each well according to the manufacturer's protocol, and the cell viability was calculated using the optical density at 450 nm and recorded using a microplate reader.

### Flow cytometry

An automated high-performance flow cytometer (FACS Arial II, BD Biosciences) was used for flow cytometric analysis to determine the percentage of apoptotic and necrotic cells. During analysis, 4T1 cells (5 × 10^3^ cells per well) were cultured in 96-well plates with PBS (control), PFC@PEPP, or PFC@PEPP-Fe and incubated for 12 h. Subsequently, the incubated cells were washed twice with PBS and incubated with anti-mouse antibodies (against Annexin V-FITC and PE) for 20 min at room temperature in the dark in PBS containing FBS (1%). Subsequently, each sample was analyzed by flow cytometry.

### Animal experiments with 4T1 tumors

All experimental animal care and handling procedures were carried out in accordance with the guidelines of the Korea Research Institute of Bioscience and Biotechnology (KRIBB) and the guidelines and policies for rodent experiments provided by the KRIBB-Institutional Animal Care and Use Committee (IACUC) (approval number: KRIBB-AEC-20152). balb/c nude mice (4-weeks-old, female) were purchased from NARA Biotech (Seoul, Korea). To generate an animal tumor model in female balb/c nude mice, 5-week-old mice were subcutaneously inoculated with 5.0 × 10^6^ 4T1 cells in serum-free RPMI 1640 medium (100 μL). Following inoculation with 4T1 cells, the mice were monitored daily until a tumor volume of ~80 mm^3^ was reached.

### Tumor volume evaluation

The size of the 4T1 tumors in the balb/c nude mice was monitored by measuring the tumor volume every 2 days using a caliper (Digimatic Caliper, Mitutoyo, Japan) until the end of the study. The tumor volume (*V*) was calculated using the following equation:


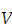
 = 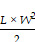


where *L* is the tumor length and *W* is the tumor width.

### *In vivo* US/FL imaging and biodistribution

*In vivo* FL images were acquired using an IVIS Lumina *in vivo* imaging system (Caliper Life Sciences) with a Cy 3.5 emission filter. For whole-body FL imaging, PFC@PEPP-Fe (0.1 mg mL^-1^, 100 μL) was administered to 4T1 tumor-bearing balb/c nude mice (6-weeks-old, female) via intravenous injection (via the tail vein). Subsequently, the mice were regularly monitored, and *in vivo*/*ex vivo* FL images were recorded using IVIS equipment. The FL intensities of the major organs and tumors extracted from the balb/c nude mice were quantified using an IVIS program (Living Image, 64-bit) for quantitative analysis. All animal experiments were carried out under isoflurane anesthesia using a gas anesthesia system (VetFloTM; Kent Scientific, USA).

For *in vivo* US imaging, 4T1 tumor-bearing balb/c nude mice (6-weeks-old, female) were intravenously injected with PBS and PFC@PEPP-Fe (100 μL, 0.1 mg mL^-1^). A B-mode US imaging system was used to collect US images of the tumor site at different time intervals (Voluson Expert 22, GE Healthcare, USA).

### *In vivo* therapeutic effect and biosafety assessment

The balb/c nude mice bearing 4T1 tumors (~80 mm^3^ in size) were randomly divided into four groups of five mice each. The mice in the control group, i.e., group (1), were intravenously injected with PBS. In contrast, the mice in groups (2) and (3) were intravenously injected with PFC@PEPP and PFC@PEPP-Fe, respectively, while the mice in group (4) were intravenously injected with PFC@PEPP-Fe and exposed to NIR laser irradiation (PFC@PEPP-Fe + NIR).

Six hours after the administration of PFC@PEPP-Fe, balb/c nude mice bearing 4T1 tumors were subjected to NIR laser irradiation (1 W cm^-^²) for 5 min. Changes in the tumor size and body weight of each mouse were continuously monitored during the 14 days treatment period.

To assess the biosafety of PFC@PEPP-Fe, peripheral blood samples were collected from the superficial temporal vein of 4T1 tumor-bearing balb/c nude mice using a 21-gauge needle at approximately 0, 1, 2, 3, 6, and 12 h post-injection. Blood iron content was subsequently quantified using ICP-MS analysis.

### Histological assessment

For histological analysis, the major organs (lungs, heart, liver, kidneys, and tumors) of the mice in the different treatment groups were excised, fixed in paraformaldehyde (4%), and embedded in paraffin. Sections with a thickness of ~3-5 μm were prepared using a microtome (HistoCore AUTOCUT, Leica Biosystems, Germany) and subjected to H&E, TUNEL, Ki-67, and CRT staining following the manufacturer's instructions.

### Statistical analysis

A t-test and analysis of variance (ANOVA) were used to determine the statistically significant differences between the control and experimental groups. All p-values less than 0.05 were marked with asterisks in the figures.

## Supplementary Material

Supplementary figures.

## Abbreviations

EPP: epinephrine
PEPP: polyepinephrine
PFC: perfluorocarbon
PFC@PEPP-Fe: PFC-encapsulated fluorescent PEPP nanoshells chelated with Fe^2+^
DA: dopamine
PDA: polydopamine
EDA: ethylenediamine
FL: fluorescence
US: ultrasound
ROS: reactive oxygen species
HTA: hydroxyterephthalic acid
CCK-8: cell counting kit-8
NIR: near-infrared
EPR: enhanced permeability and retention
DLS: dynamic light scattering
FT-IR: Fourier-transform infrared spectroscopy
TEM: transmission electron microscopy
EDS: energy-dispersive X-ray spectroscopy
XPS: X-ray photoelectron spectroscopy
ICP-MS: inductively coupled plasma mass spectroscopy
H&E: hematoxylin and eosin
TUNEL: a terminal deoxynucleotidyl transferase biotin-dUTP nick end labeling
CRT: calreticulin
