# Supplementary material for: Perfluorocarbon-polyepinephrine core-shell nanoparticles as a near-infrared light activatable theranostic platform for bimodal imaging-guided photothermal/chemodynamic synergistic cancer therapy
Source: Theranostics. 2025 Jan 1;15(3):1077–93. doi: 10.7150/thno.102743 (PMC11700858; doi:10.7150/thno.102743)
Supplement: Supplementary file 1 — Supplementary figures. [file thnov15p1077s1.pdf]

Supporting Information

Perfluorocarbon-polyepinephrine core-shell nanoparticles as a near-infrared light activatable theranostic platform for bimodal imaging-guided photothermal/chemodynamic synergistic cancer therapy

Kyung Kwan Lee[1], Kyung-Woo Park[1], Sang Cheon Lee[2,*] and Chang-Soo Lee[1,3,*]

1. Bionanotechnology Research Center, Korea Research Institute of Bioscience and Biotechnology (KRIBB), Daejeon 34141, Republic of Korea
2. Department of Maxillofacial Biomedical Engineering, School of Dentistry, Kyung Hee University, Seoul 02447, Republic of Korea
3. Department of Biotechnology, University of Science & Technology (UST), Daejeon 34113, Republic of Korea

*Corresponding authors: Prof. Sang Cheon Lee, email: schlee@khu.re.kr; Dr. Chang-Soo Lee, email: cslee@kribb.re.kr

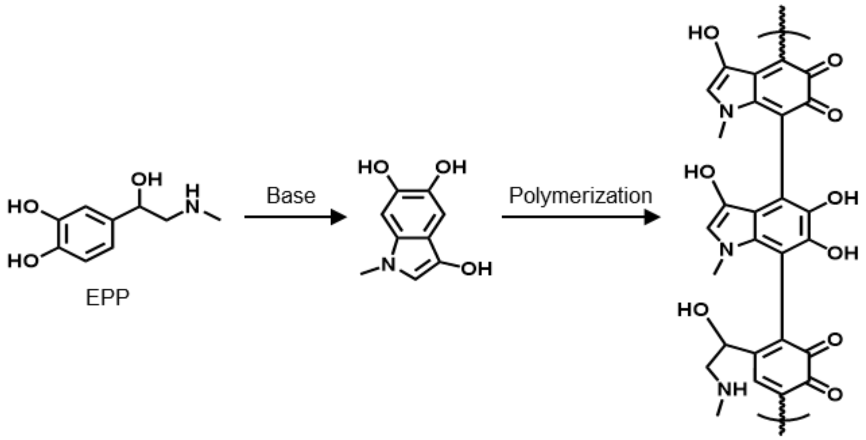

Figure S1. General scheme illustrating the formation process of PEPP through the oxidation of EPP.

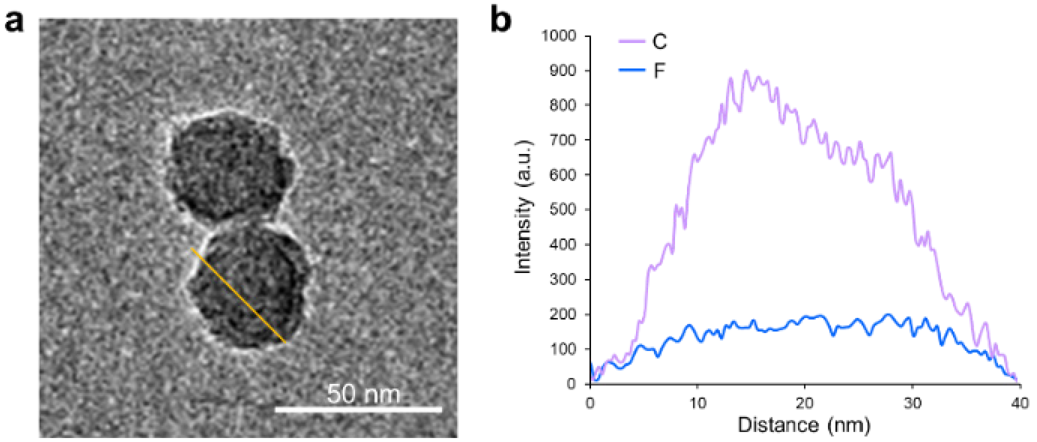

Figure S2. TEM image of PFC@PEPP with a yellow line indicating the EDS line scan position and (b) EDS line scan with carbon (C) and fluorine (F) signals, respectively.

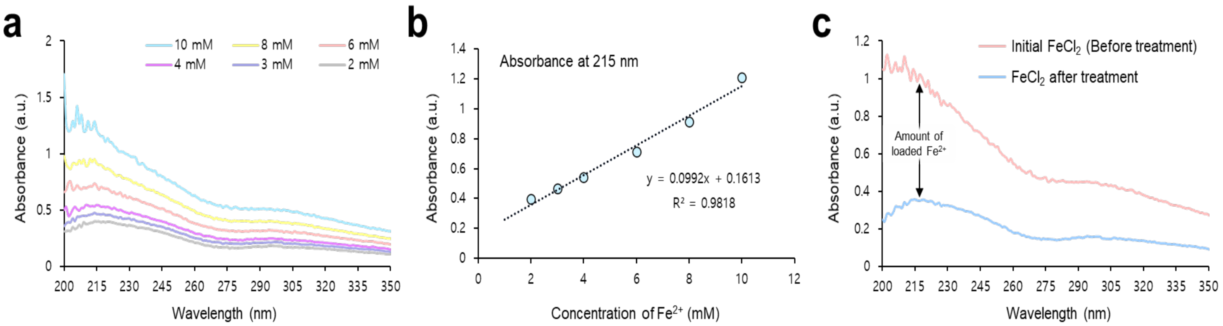

Figure S3. (a) UV-Vis spectrum and (b) corresponding calibration curve of $FeCl_2$ at various concentrations. (c) UV-Vis spectrum of $FeCl_2$ after and before treatment of PFC@PEPP.

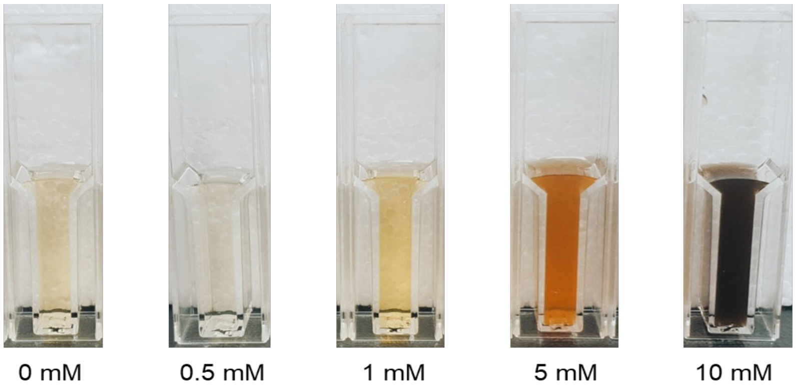

Figure S4. Camera images of PFC@PEPP with varying concentrations of Fe^{2+}.

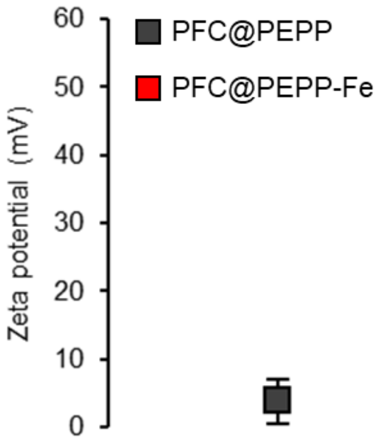

Figure S5. Zeta-potential value of PFC@PEPP and PFC@PEPP-Fe.

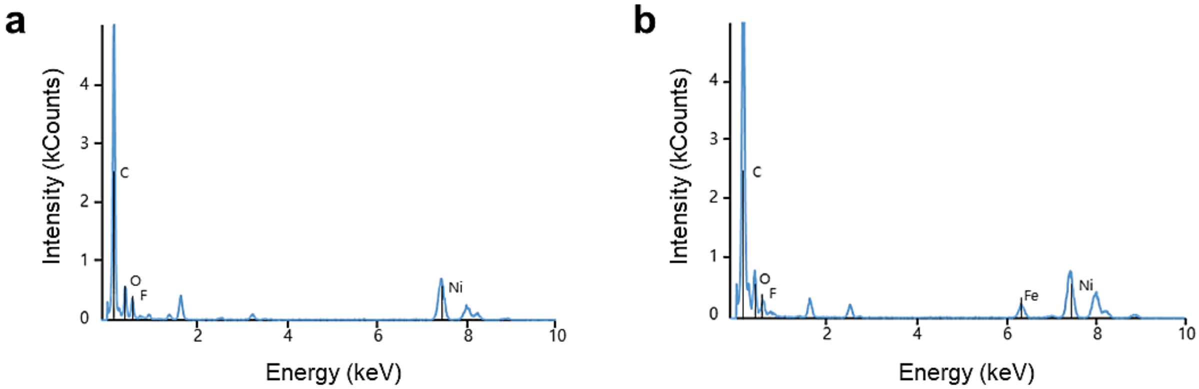

Figure S6. TEM-associated EDS spectra of (a) PFC@PEPP and (b) PFC@PEPP-Fe. (The utilization of a nickel grid is the cause of the Ni peak.)

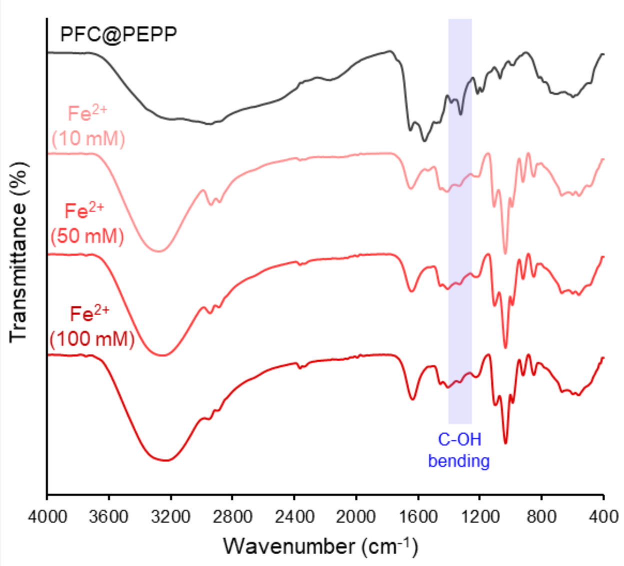

Figure S7. FT-IR spectra of PFC@PEPP and PFC@PEPP-Fe prepared with the addition of $FeCl_2$ at various concentrations.

	F1s	Fe2p
PFC@PEPP	4.34	0.52
PFC@PEPP-Fe	3.01	9.76

Figure S8. Elemental composition analysis of PFC@PEPP and PFC@PEPP-Fe.

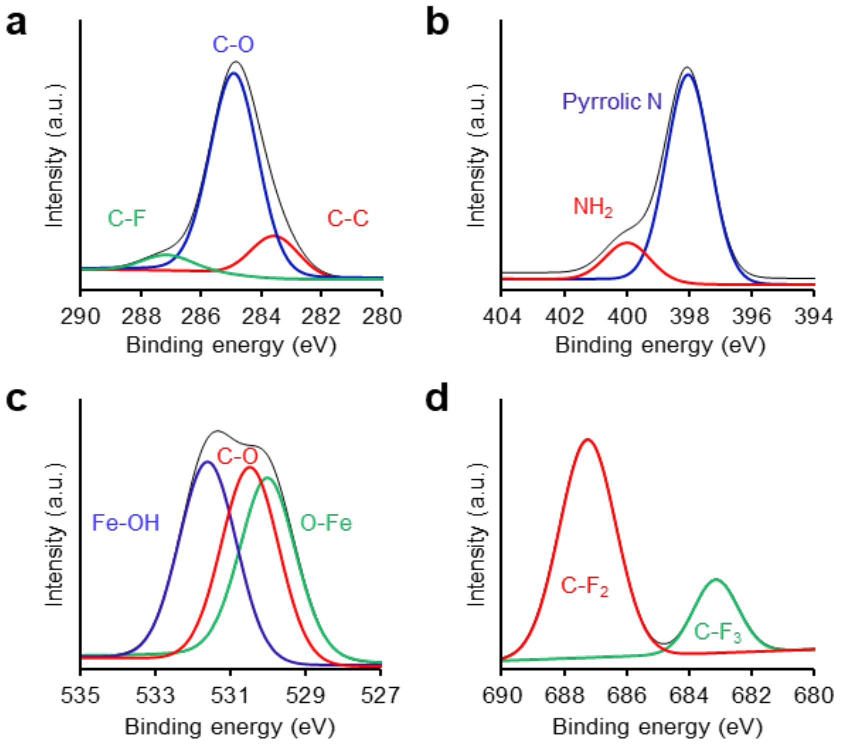

Figure S9. XPS narrow scan spectra of PFC@PEPP-Fe, including (a) C1s, (b) N1s, (c) O1s, and (d) F 1s.

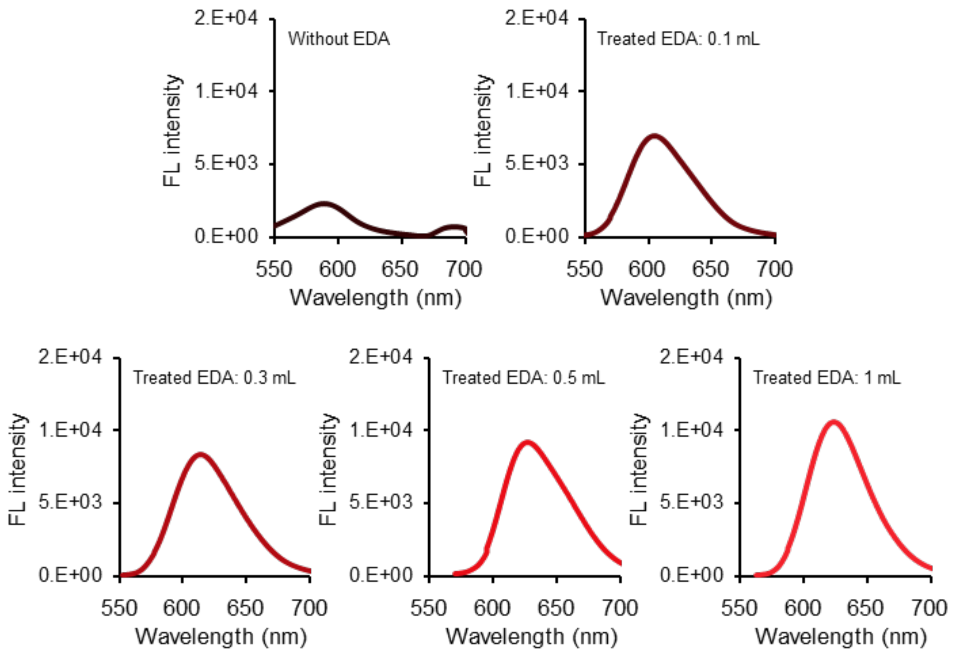

Figure S10. FL emission spectrum of PFC@PEPP prepared with addition of EDA at various concentrations (excitation at 480 nm).

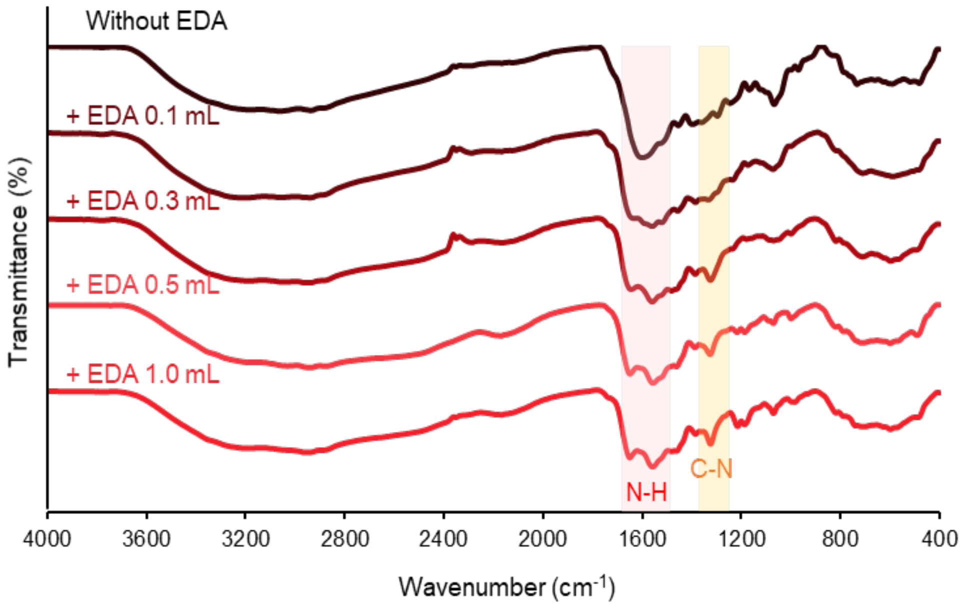

Figure S11. FT-IR spectra of PFC@PEPP indicating N-H and C-N associations, prepared with the addition of EDA at various concentrations.

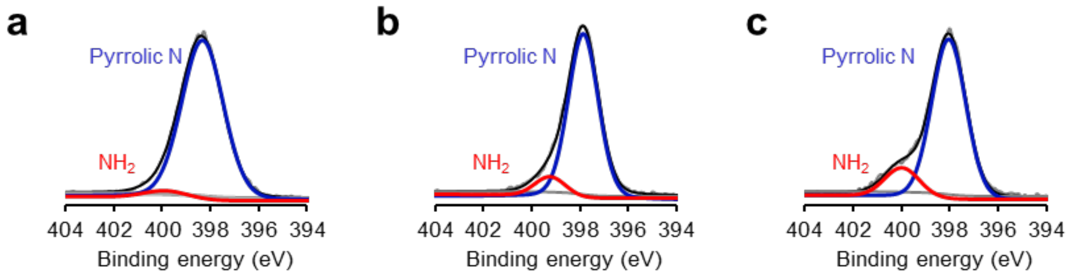

Figure S12. XPS narrow scan spectra indicating the NH_2 and pyrrolic N binding energies for N 1s of PFC@PEPP with (a) 0.1 mL, (b) 0.5 mL and (c) 1 mL of EDA added.

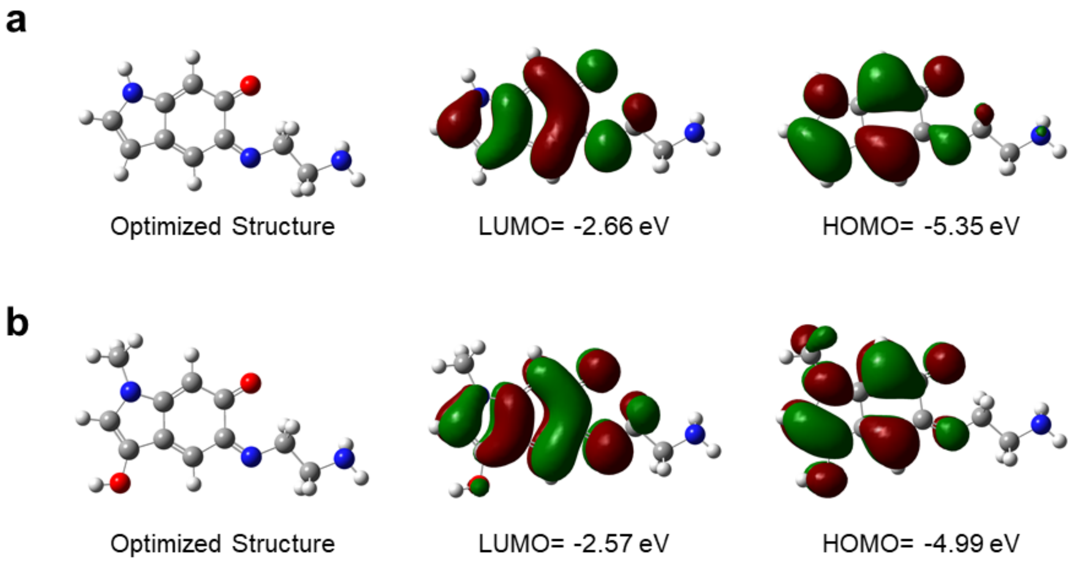

Figure S13. Theoretical calculated molecular orbitals of (a) fluorescent polydopamine NPs synthesized from DA monomer and (b) fluorescent PEPP synthesized from EPP monomer using DFT calculations at the B3LYP/3-31G (d,p) level.

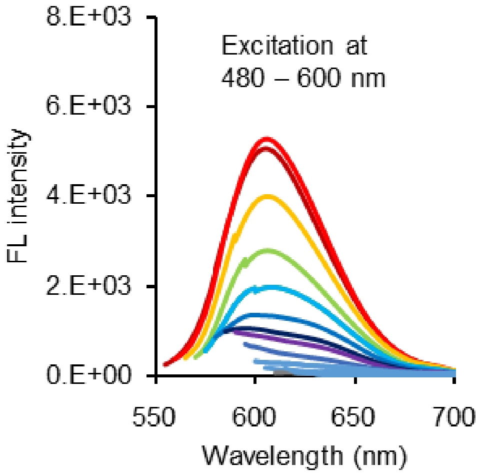

Figure S14. FL emission spectra of PFC@PEPP at different excitation wavelengths.

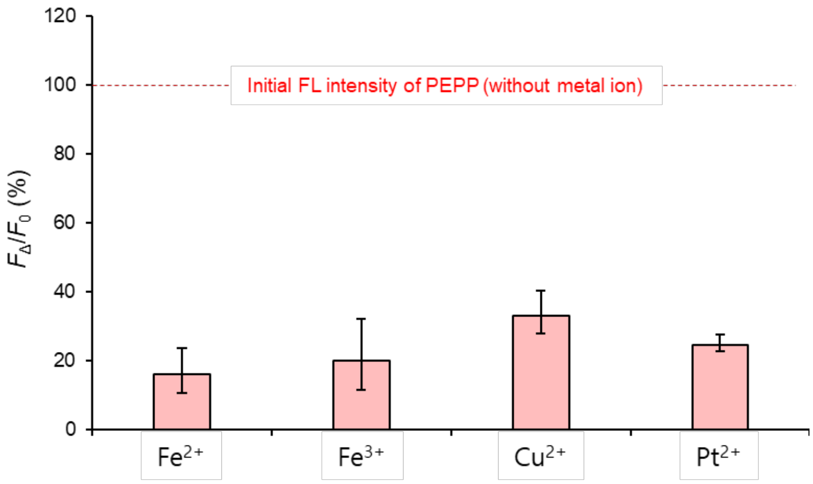

Figure S15. FL quenching ratio of PFC@PEPP in the presence of various theranostic metal ions (10 mM).

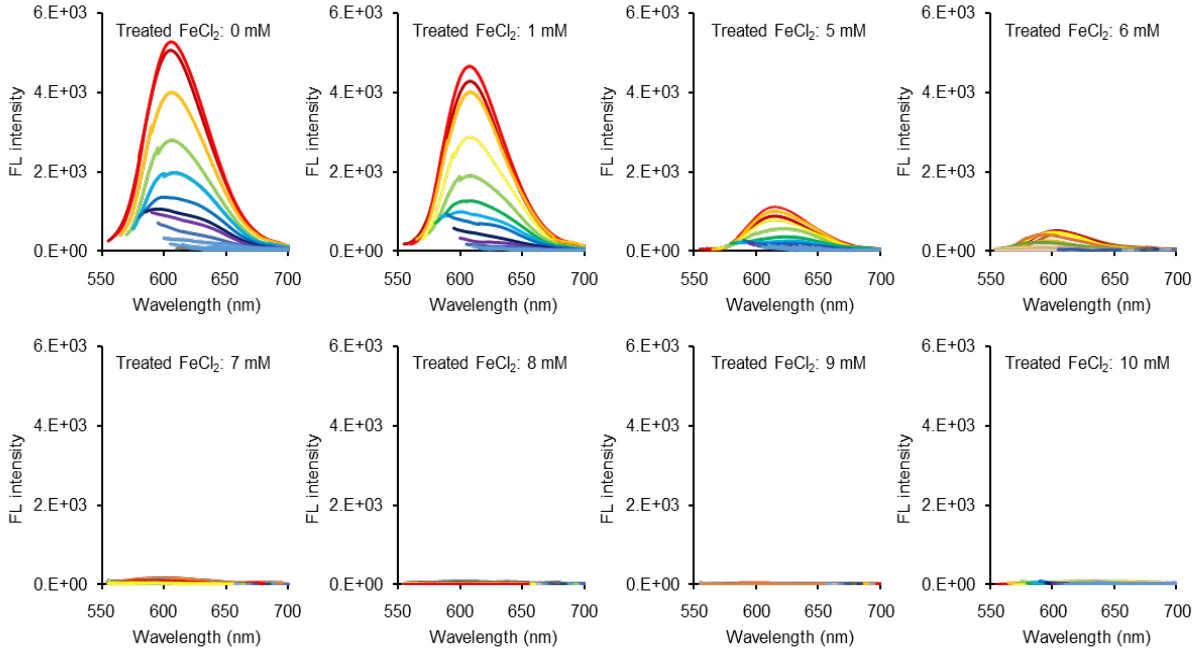

Figure S16. FL emission spectrum of PFC@PEPP after treatment with various concentrations of $FeCl_2$.

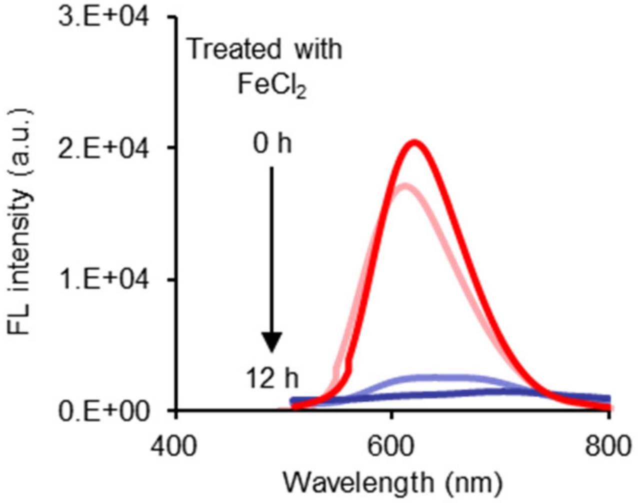

Figure S17. FL change of PFC@PEPP after treatment with $FeCl_2$ for at different time point.

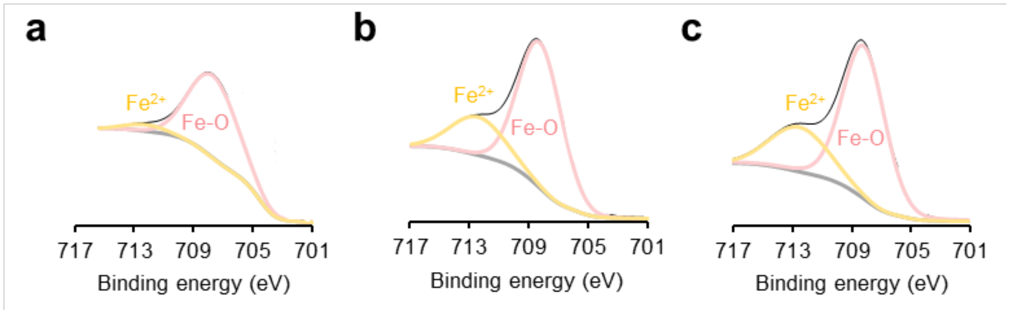

Figure S18. XPS narrow scan spectra for Fe 2p of PFC@PEPP-Fe at (a) 0 h, (b) 6 h, and (c) 12 h under pH 5.4 buffer (0.1 M sodium acetate).

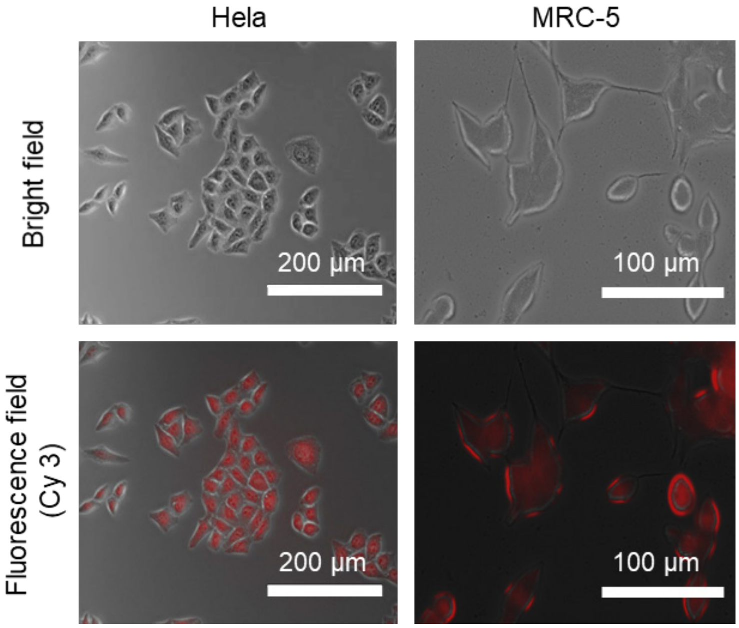

Figure S19. Intracellular FL images of cancer cells (Hela) and normal cells (MRC-5) incubated with PFC@PEPP for 12 h.

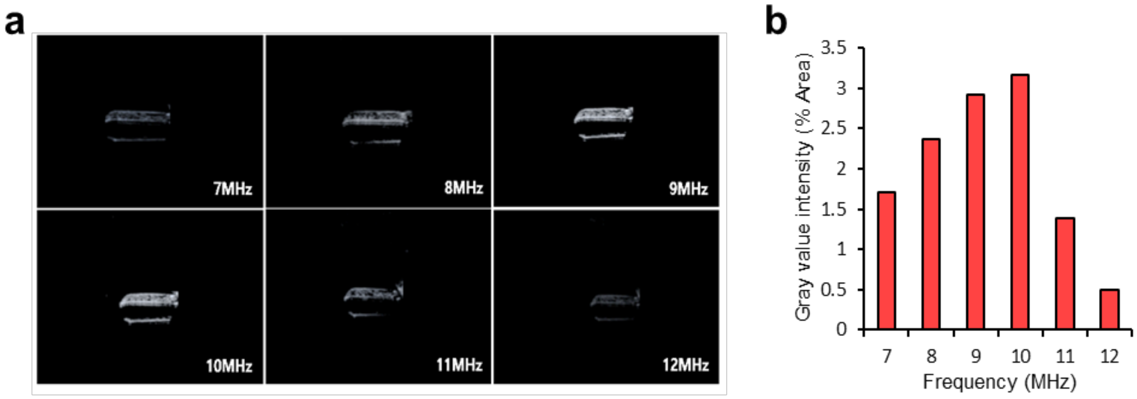

Figure S20. (a) In vitro US images of PFC@PEPP-Fe and (b) their corresponding gray values at different frequencies (10 min, at 37 °C).

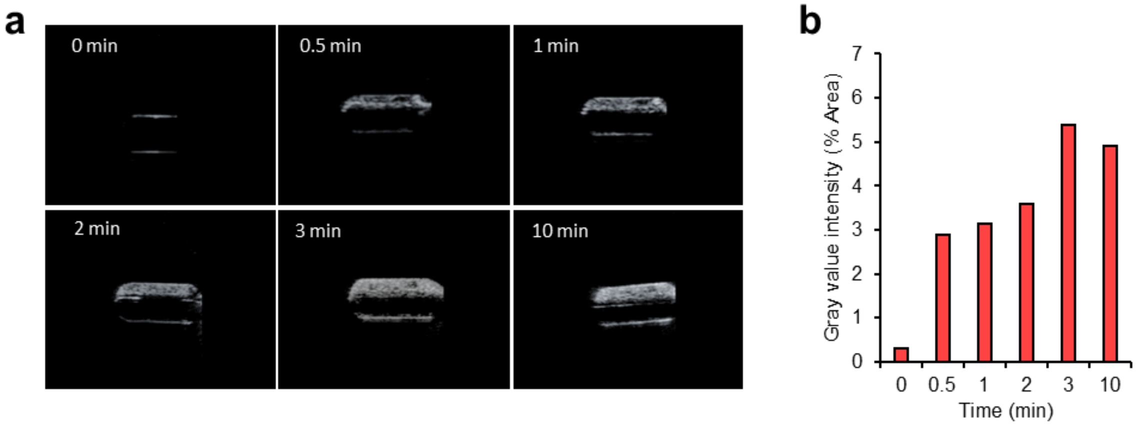

Figure S21. (a) In vitro US images of PFC@PEPP-Fe and (b) their corresponding gray values at different time intervals (10 MHz, at 37 °C).

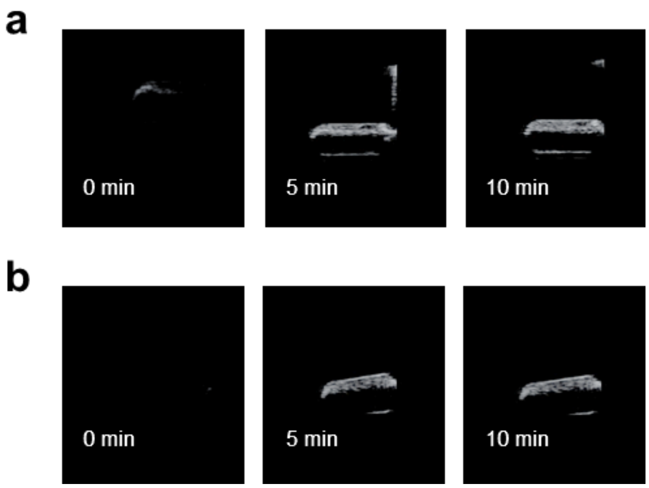

Figure S22. In vitro US images of (a) PFC@PEPP and (b) PFC@PEPP-Fe at different time intervals (10 MHz at 37 °C).

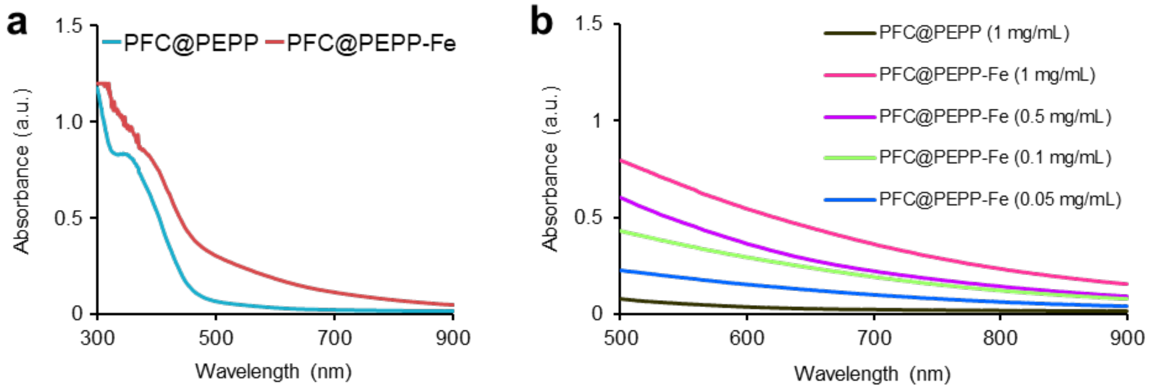

Figure S23. (a) UV-Vis spectra of PFC@PEPP and PFC@PEPP-Fe, and (b) their UV-Vis spectra in the NIR region at varying concentrations.

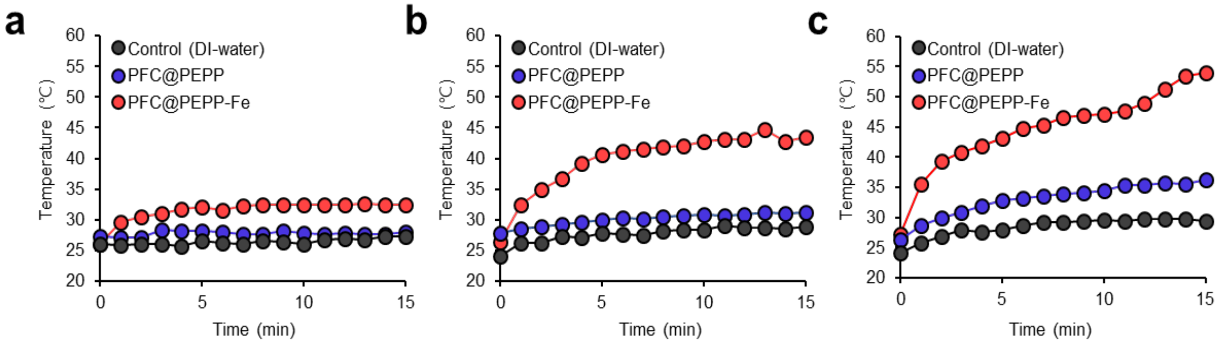

Figure S24. Time-dependent temperature variation of DI water, PFC@PEPP, and PFC@PEPP-Fe under NIR laser irradiation at different power levels of (a) 0.1 W cm², (b) 0.5 W cm², and (c) 1 W cm² for a duration of 15 min.

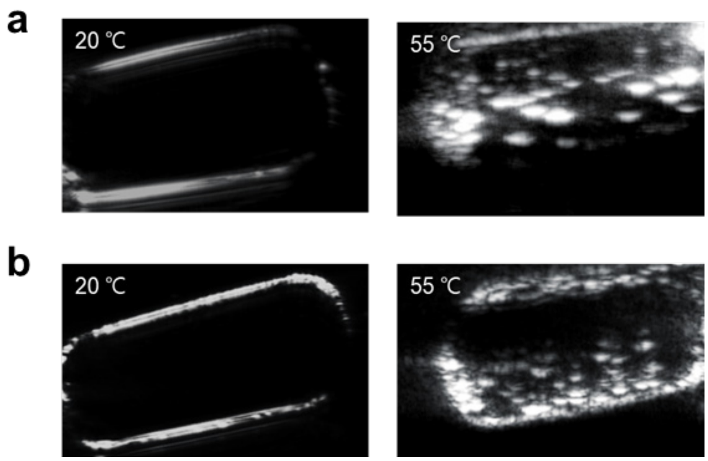

Figure S25. Temperature-dependent US images of (a) PFC@PEPP and (b) PFC@PEPP-Fe.

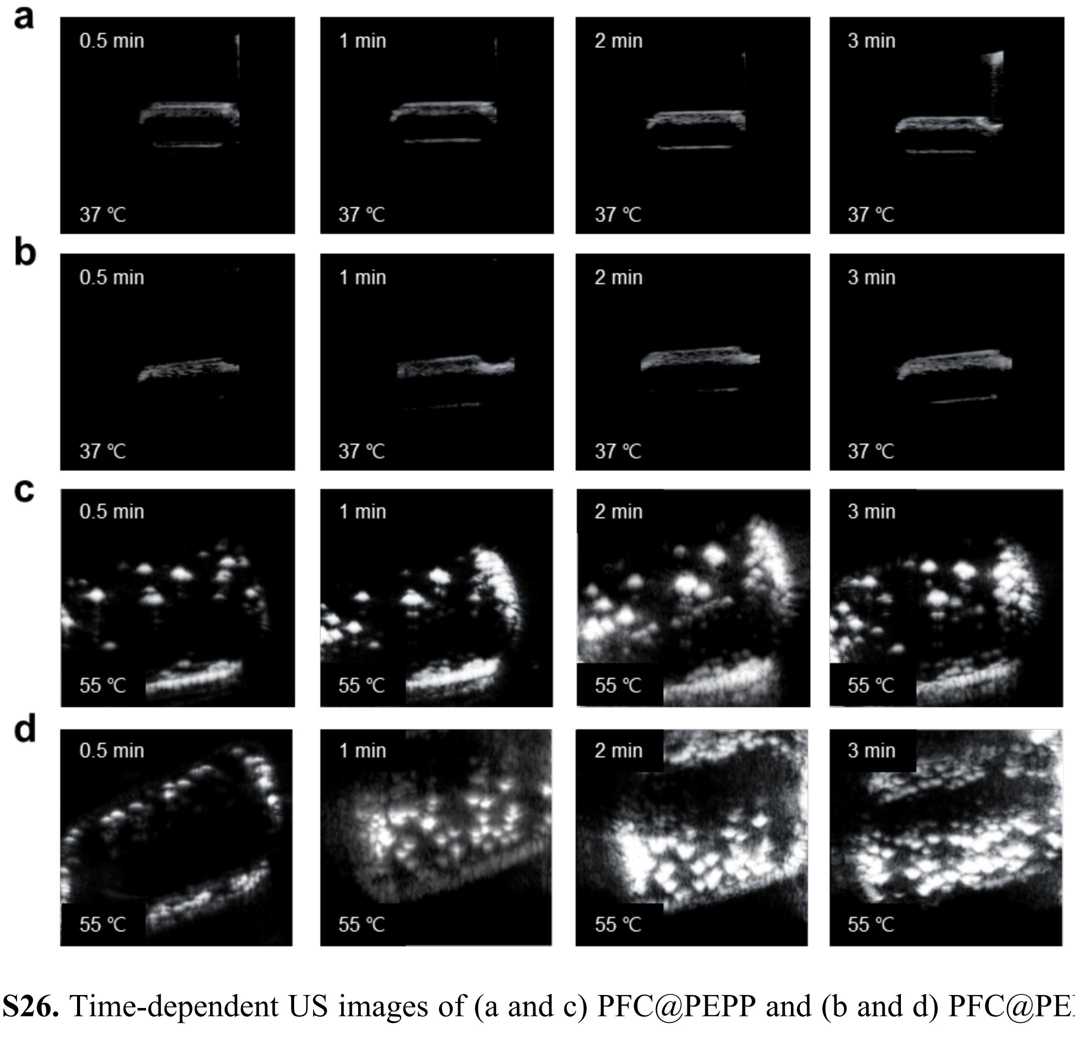

Figure S26. Time-dependent US images of (a and c) PFC@PEPP and (b and d) PFC@PEPP-Fe at 37 ℃ and 55 ℃.

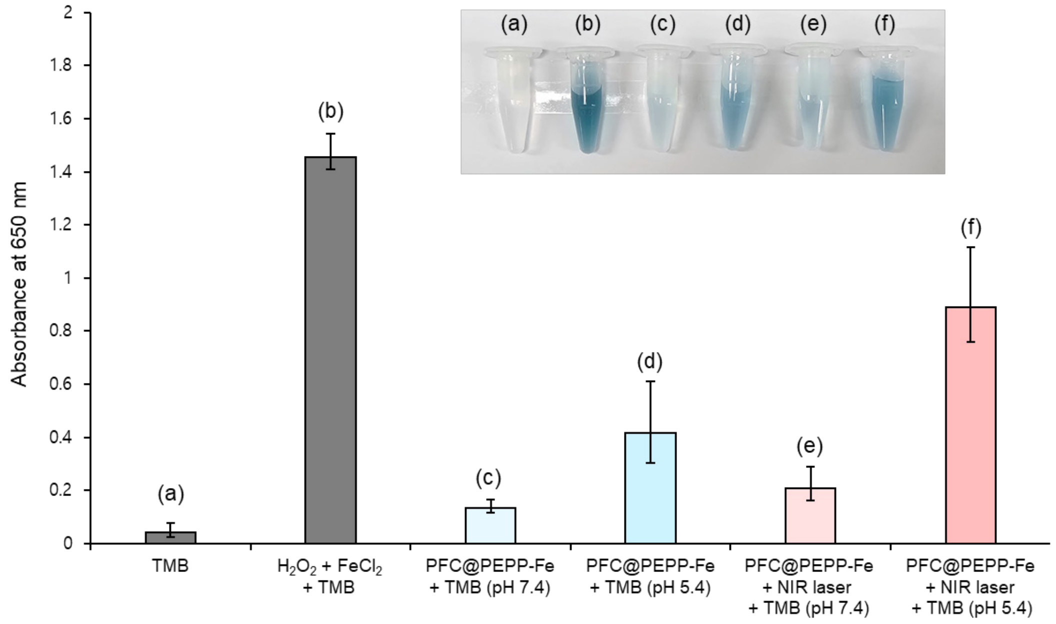

Figure S27. Absorbance of various sample groups at 650 nm.

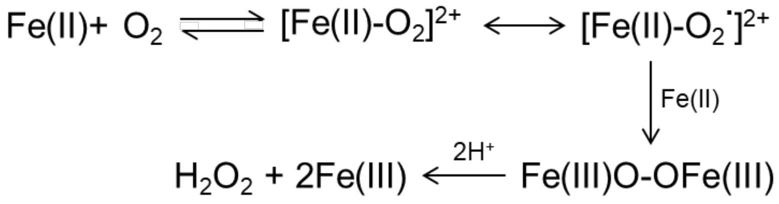

Figure S28. Proposed mechanism of the reduction process of Fe^{2+} and O_2 for H_2O_2 production.

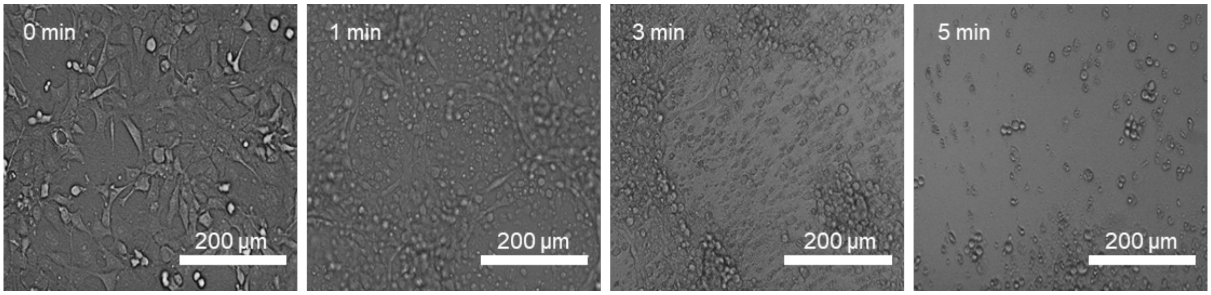

Figure S29. Optical microscopic images of 4T1 cells treated with PFC@PEPP-Fe under NIR laser irradiation (1.0 W cm^{-2}) at different time.

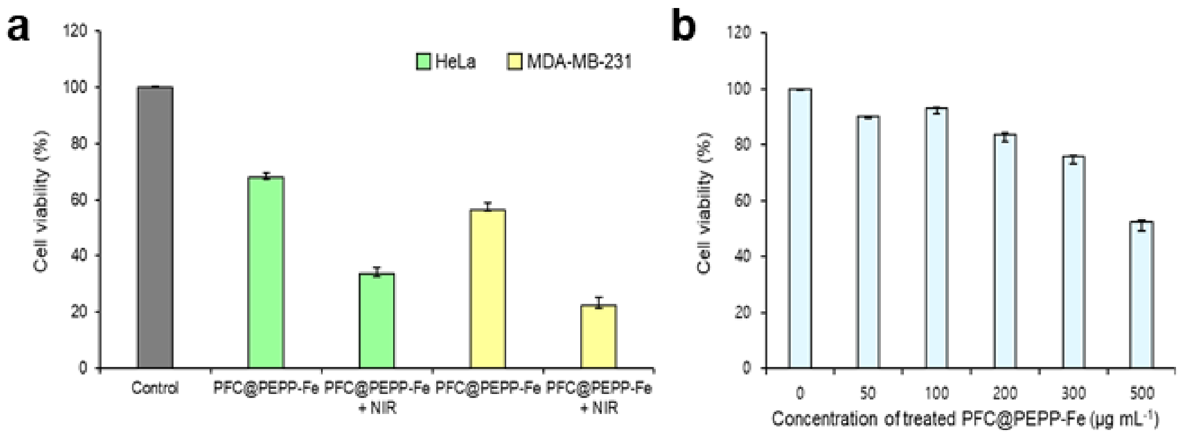

Figure S30. Anticancer effect of PFC@PEPP-Fe under with or without NIR laser irradiation on HeLa and MDA-MB-231 cells. (b) Cell viability of MRC-5 cells treated with different concentrations of PFC@PEPP-Fe.

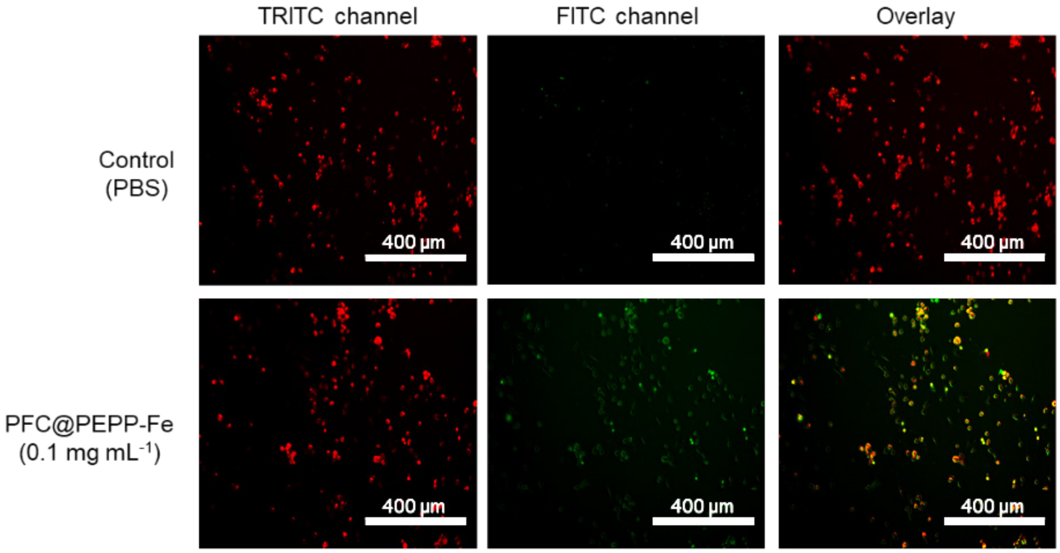

Figure S31. Fluorescence images of 4T1 cells with and without PFC@PEPP-Fe treatment, observed using the Lipid Peroxidation Assay Kit.

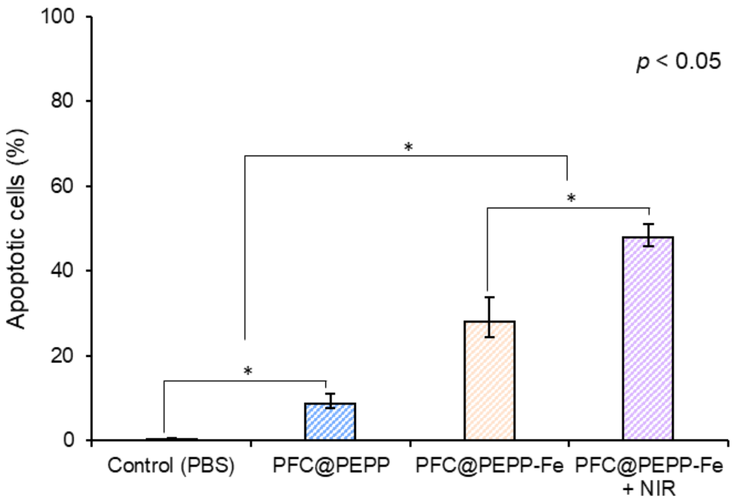

Figure S32. Proportion of apoptotic cells in 4T1 cells treated with PBS, PFC@PEPP, PFC@PEPP-Fe, and PFC@PEPP-FE + NIR.

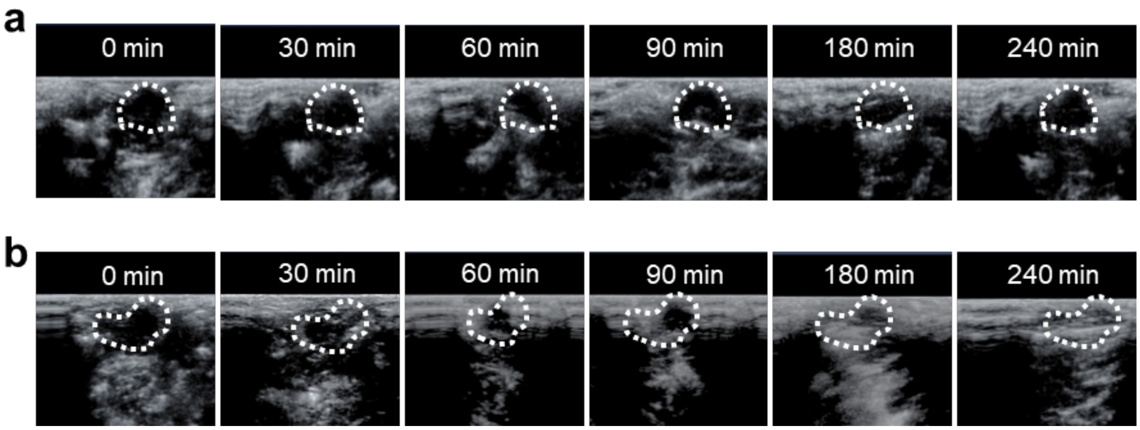

Figure S33. Time-dependent US images of 4T1 tumor-bearing balb/c nude mice at the tumor site (white dotted line), treated intravenously with (a) PBS and (b) PFC@PEPP-Fe.

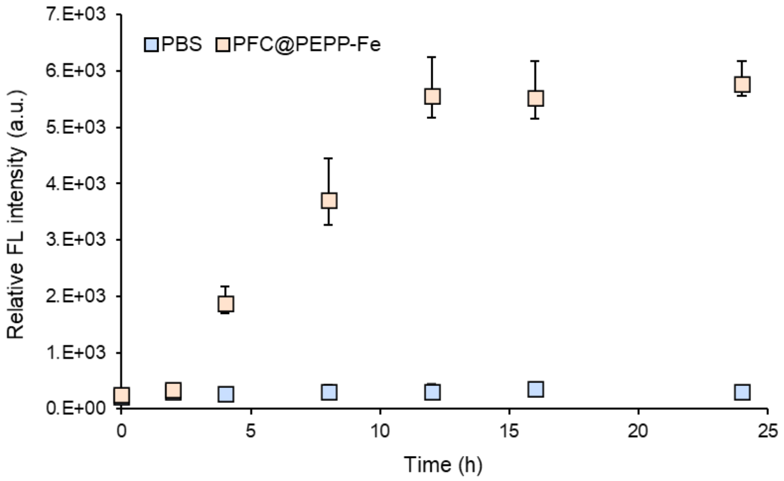

Figure S34. Time-dependent FL intensity at the tumor site following intravenous injection of PBS and PFC@PEPP-Fe after biodistribution analysis.

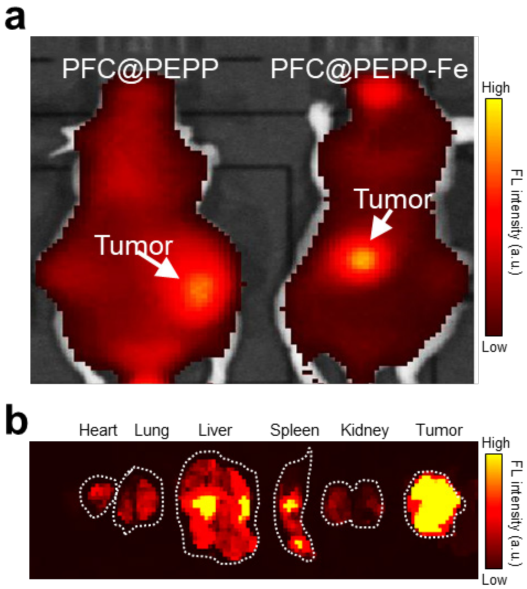

Figure S35. (a) FL image captured 24 h after intravenous injection of PFC@PEPP and PFC@PEPP-Fe in 4T1 tumor-bearing balb/nude mice. (b) FL images of the heart, lung, liver, spleen, kidney, and tumor excised from balb/c nude mice 24 h after intravenous injection of PFC@PEPP.

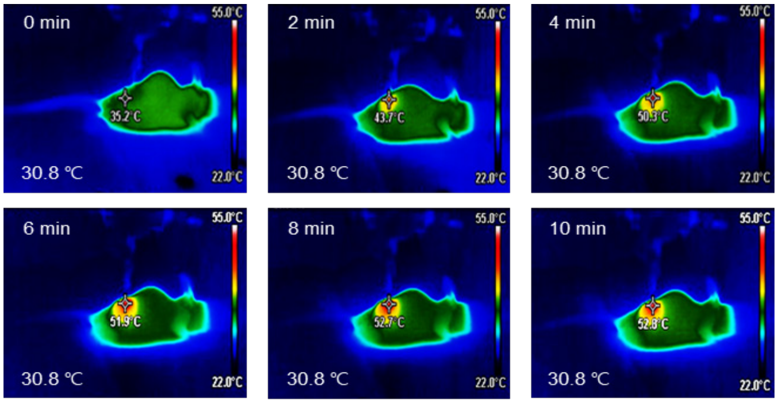

Figure S36. Time-dependent IR thermal images of 4T1 tumor-bearing balb/c nude mice obtained after intravenous injection of PFC@PEPP-Fe, followed by exposure to an 808 nm laser (1.0 W cm^{-2}) for 10 min.

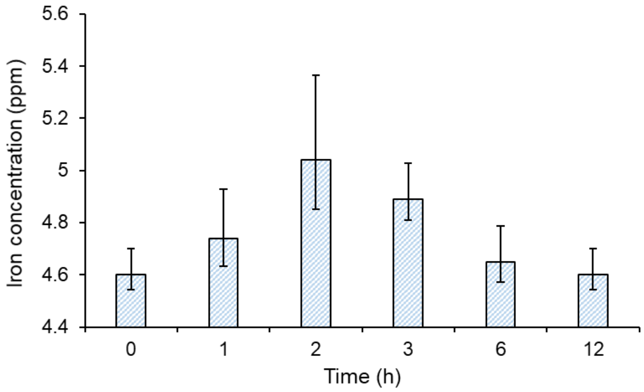

Figure S37. Iron concentration of peripheral blood within 4T1 tumor-bearing balb/c nude mice after intravenous injected with PFC@PEPP-Fe at different time point.

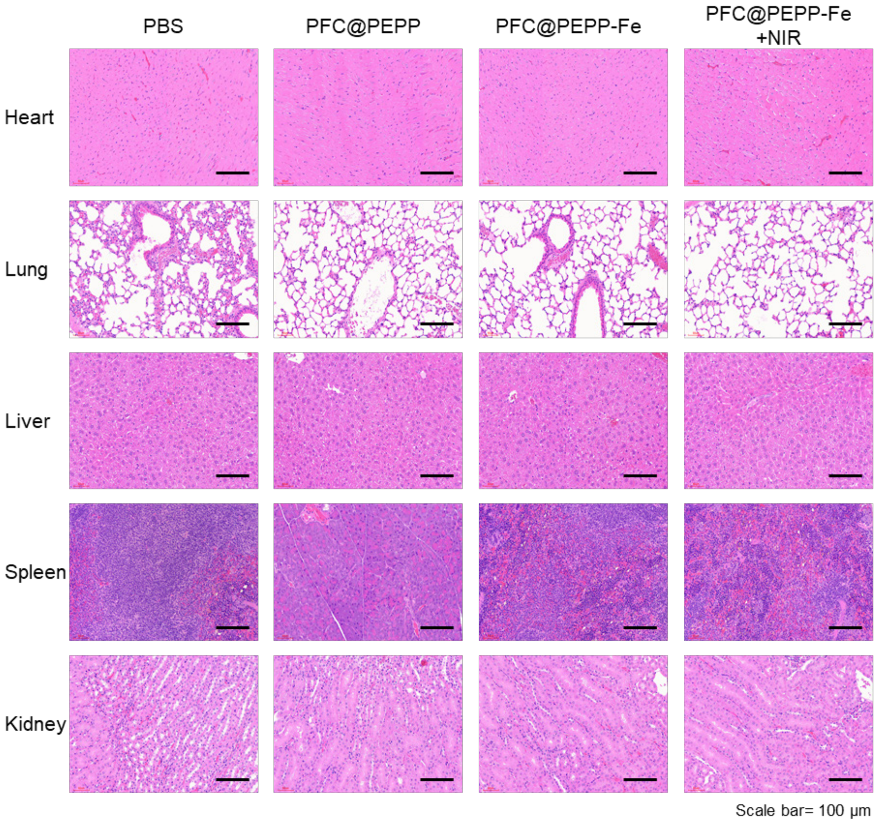

Figure S38. H&E stained images of the heart, lung, liver, spleen, and tumor obtained from 4T1 tumor-bearing balb/c nude mice in the various treated groups after a treatment duration of 14 days.
